# Altered Distribution of RNA Polymerase Lacking the Omega Subunit within the Prophages along the *Escherichia coli* K-12 Genome

**DOI:** 10.1128/mSystems.00172-17

**Published:** 2018-02-13

**Authors:** Kaneyoshi Yamamoto, Yuki Yamanaka, Tomohiro Shimada, Paramita Sarkar, Myu Yoshida, Neerupma Bhardwaj, Hiroki Watanabe, Yuki Taira, Dipankar Chatterji, Akira Ishihama

**Affiliations:** a Department of Frontier Bioscience, Hosei University, Tokyo, Japan; b Micro-Nano Technology Research Center, Hosei University, Tokyo, Japan; c Meiji University, School of Agriculture, Kawasaki, Kanagawa, Japan; d Indian Institute of Science, Molecular Biophysics Unit, Bangalore, India; University of California, Berkeley

**Keywords:** *Escherichia coli*, RNA polymerase, omega subunit, prophage, transcription regulation

## Abstract

The RNA polymerase (RNAP) of *Escherichia coli* K-12 is a complex enzyme consisting of the core enzyme with the subunit structure α_2_ββ′ω and one of the σ subunits with promoter recognition properties. The smallest subunit, omega (the *rpoZ* gene product), participates in subunit assembly by supporting the folding of the largest subunit, β′, but its functional role remains unsolved except for its involvement in ppGpp binding and stringent response. As an initial approach for elucidation of its functional role, we performed in this study ChIP-chip (chromatin immunoprecipitation with microarray technology) analysis of wild-type and *rpoZ*-defective mutant strains. The altered distribution of RpoZ-defective RNAP was identified mostly within open reading frames, in particular, of the genes inside prophages. For the genes that exhibited increased or decreased distribution of RpoZ-defective RNAP, the level of transcripts increased or decreased, respectively, as detected by reverse transcription-quantitative PCR (qRT-PCR). In parallel, we analyzed, using genomic SELEX (systemic evolution of ligands by exponential enrichment), the distribution of constitutive promoters that are recognized by RNAP RpoD holoenzyme alone and of general silencer H-NS within prophages. Since all 10 prophages in *E. coli* K-12 carry only a small number of promoters, the altered occupancy of RpoZ-defective RNAP and of transcripts might represent transcription initiated from as-yet-unidentified host promoters. The genes that exhibited transcription enhanced by RpoZ-defective RNAP are located in the regions of low-level H-NS binding. By using phenotype microarray (PM) assay, alterations of some phenotypes were detected for the *rpoZ*-deleted mutant, indicating the involvement of RpoZ in regulation of some genes. Possible mechanisms of altered distribution of RNAP inside prophages are discussed.

**IMPORTANCE** The 91-amino-acid-residue small-subunit omega (the *rpoZ* gene product) of *Escherichia coli* RNA polymerase plays a structural role in the formation of RNA polymerase (RNAP) as a chaperone in folding the largest subunit (β′, of 1,407 residues in length), but except for binding of the stringent signal ppGpp, little is known of its role in the control of RNAP function. After analysis of genomewide distribution of wild-type and RpoZ-defective RNAP by the ChIP-chip method, we found alteration of the RpoZ-defective RNAP inside open reading frames, in particular, of the genes within prophages. For a set of the genes that exhibited altered occupancy of the RpoZ-defective RNAP, transcription was found to be altered as observed by qRT-PCR assay. All the observations here described indicate the involvement of RpoZ in recognition of some of the prophage genes. This study advances understanding of not only the regulatory role of omega subunit in the functions of RNAP but also the regulatory interplay between prophages and the host *E. coli* for adjustment of cellular physiology to a variety of environments in nature.

## Abstract (precis)

The 91-amino-acid-residue small-subunit omega (the *rpoZ* gene product) of *Escherichia coli* RNA polymerase plays a structural role in the formation of RNA polymerase (RNAP) as a chaperone in folding the largest subunit (β′, of 1,407 residues in length), but except for binding of the stringent signal ppGpp, little is known of its role in the control of RNAP function. After analysis of genomewide distribution of wild-type and RpoZ-defective RNAP by the ChIP-chip method, we found alteration of the RpoZ-defective RNAP inside open reading frames, in particular, of the genes within prophages. For a set of the genes that exhibited altered occupancy of the RpoZ-defective RNAP, transcription was found to be altered as observed by qRT-PCR assay. All the observations here described indicate the involvement of RpoZ in recognition of some of the prophage genes. This study advances understanding of not only the regulatory role of omega subunit in the functions of RNAP but also the regulatory interplay between prophages and the host *E. coli* for adjustment of cellular physiology to a variety of environments in nature.

## INTRODUCTION

RNA polymerase (RNAP) is the key enzyme of transcription. In *Escherichia coli*, the RNAP core enzyme is composed of four subunits, α (RpoA), β (RpoB), β′ (RpoC), and ω (RpoZ), in the stoichiometry of α_2_ββ′ω (reviewed in references 1 and 2. The core enzyme is assembled sequentially in the order 2α > α_2_ > α_2_β > α_2_ββ′ω (3, 4). In this assembly pathway, ω interacts with β′, forming β′ω intermediate (5), which then binds to the preformed α_2_β complex, leading to formation of the core enzyme. A model of chaperone function was proposed for 91-residue-long ω (RpoZ) in supporting the folding of the longest polypeptide, β′ (RpoC), of 1,407 residues in size. RNAP purified from the *rpoZ*-defective mutant is associated with GroL, indicating the participation of GroL chaperone in place of ω in the process of RNAP formation (6). In agreement with this assembly mechanism of RNAP, the *rpoZ* gene is not an essential gene and the mutant lacking *rpoZ* is viable (7). In the core enzyme, RpoZ is present in near-stoichiometric amounts with respect to other subunits (8). The crystal structures of RNAP from *Thermus aquaticus* (9) and *E. coli* (10) confirm that RpoZ is one of the RNAP subunits.

Structure-function relationships have been extensively characterized for RpoA, RpoB, and RpoC (11, 12), but except for the structural role in assembly of RNAP, the functional role played by RpoZ remains unsolved (13). When *E. coli* RNAP is associated with a dominant negative variant of RpoZ subunit, the resulting RNAP is defective in initiation of transcription, although preinitiated RNAP-RNA complex can elongate transcription (14). When RpoZ is tethered to DNA-binding protein, it is able to activate transcription through protein-protein contact between RNAP and the RpoZ segment (15). These findings indicate the direct molecular contact between RpoZ and other subunits of RNAP. Several lines of observation indicated the involvement of RpoZ in the functional control of RNAP (14, 16). A functional link between the RpoZ subunit and the stringent response has been elucidated: first, *in vitro* transcription by purified RNAP without RpoZ subunit was found to be insensitive to ppGpp (17), and the RpoZ-less RNAP regains its sensitivity to (p)ppGpp upon the external addition of the RpoZ subunit (18) or the protein DksA, a collaborative player in the stringent response (19). Direct binding of ppGpp at a site near the rifampin binding site on the RpoB subunit was indicated by genetic and cross-linking studies (20–22), while the recent crystal structure of RNAP-ppGpp complex and mutational studies on *E. coli* RNAP showed that ppGpp binds at the interface of RpoC and RpoZ subunit (23–25). The level of RpoZ subunit influences DNA relaxation in *E. coli* (26), implying difference in DNA-binding properties between wild-type RNAP and RpoZ-defective RNAP. Microarray analysis of transcriptome in the absence of RpoZ indicated alteration in transcription of a set of genes, including the *relA* gene encoding ppGpp synthetase (7). These observations altogether indicate that the function of RNAP is controlled by the associated RpoZ subunit. Besides the ppGpp-binding site on the RpoZ subunit (ppGpp site 1), ppGpp binding was recently identified at an interface between RNAP and DksA (ppGpp site 2) (27). In the stringent control, a small transcription factor, DksA, participates in conjunction with ppGpp (19).

In this study, we made attempts to find the regulatory role of RpoZ subunit in the function of RNAP and transcription. As a shortcut approach to get insights into the overall functional role of RpoZ subunit in the transcription of the *E. coli* genome, we have performed, in this study, ChIP-chip (chromatin immunoprecipitation with microarray technology) analysis of the distribution of RNAP along the genome in the presence and absence of RpoZ subunit. Results of the ChIP-chip analysis indicated that the distribution of RNAP lacking RpoZ in growing *E. coli* K-12 cells is altered compared with that of wild-type RNAP mostly in the middle of the open reading frame (ORF) of a specific set of target genes. Surprisingly, the majority of these genes are located within the cryptic prophages. This finding was confirmed by measuring mRNA for these genes. These unexpected findings raise an interesting possibility of the involvement of RpoZ in the recognition of prophage genes. This could make a breakthrough in the identification of the functional role of RpoZ.

## RESULTS

### Altered distribution of RpoZ-defective RNAP inside the prophages along the *E. coli* genome.

To examine the possible influence of the presence and absence of RpoZ on the distribution of RNAP along the genome, we performed ChIP-chip analysis (28, 29) for *E. coli* K-12 wild-type strain BW25113 and its *rpoZ*-deleted mutant JW3624 from the Keio collection (30). The absence of RpoZ in JW3624 was confirmed by Western blotting analysis using specific anti-RpoZ antibody (see Fig. S1 in the supplemental material). In a synthetic M9-glucose medium, JW3624 showed a similar growth pattern as that of the parent strain BW25113 until the early stationary phase, supporting the concept that the *rpoZ* gene is not essential and does not influence cell growth. The growth patterns differed between the two strains after prolonged incubation (see below).

10.1128/mSystems.00172-17.1 FIG S1  The absence of RpoZ in *E. coli* JW3624. *E. coli* K-12 BW25113 (wild type) and its *rpoZ*-defective mutant strain, JW3624, were grown in LB medium until logarithmic phase. Cell lysates were prepared and subjected to Western blotting using anti-RpoA, anti-RpoB, anti-RpoC, and anti-RpoZ. Download FIG S1, PDF file, 0.4 MB. Copyright © 2018 Yamamoto et al. 2018 Yamamoto et al. This content is distributed under the terms of the Creative Commons Attribution 4.0 International license.

In the middle of exponential growth phase, both wild-type BW25113 and *rpoZ*-defective JW3624 strains were treated with formaldehyde at a final concentration of 1% for cross-linking between proteins and genomic DNA. After 30 min, lysates were prepared, sonicated for DNA fragmentation, and then subjected to immunoprecipitation with anti-RpoA antibody. RNAP-conjugated DNAs in immunoprecipitates were digested by pronase, and then free DNA fragments were purified using a QIAquick PCR purification kit (Qiagen). Recovered DNA fragments were amplified by PCR using a pair of random primers. DNA from wild-type BW25113 was labeled with Cy3, while that from *rpoZ*-defective JW3624 was labeled with Cy5. The DNA mixture was subjected to tiling array analysis for mapping DNA segments along the *E. coli* genome. The ratio of Cy3 and Cy5 fluorescence intensity bound to each probe was plotted along the *E. coli* K-12 genome, and thus, the relative intensity of Cy3/Cy5 represents the relative distribution between wild-type and RpoZ-defective mutant RNAP at each probe position. RNAP-bound DNA fragments of about 250 bp in size should bind to two or more 60-bp-long probes aligned at 105-bp intervals, and thus, a single peak was estimated to be a background noise.

**FIG 1:**
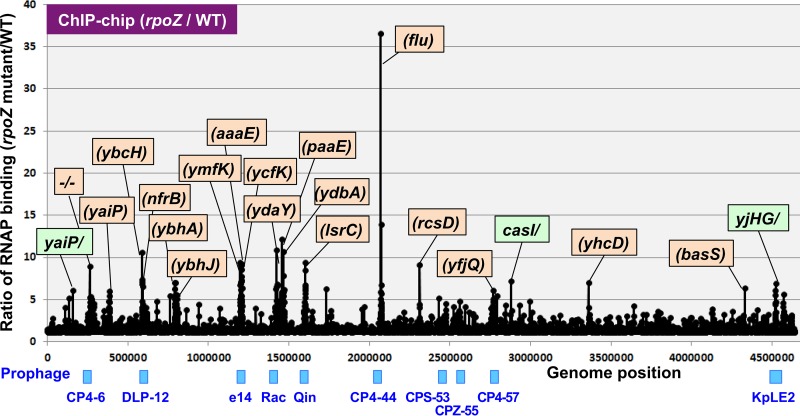
ChIP-chip analysis of wild-type (WT) and RpoZ-defective mutant RNAP. The ChIP-chip analysis was performed for *E. coli* K-12 wild-type BW25113 and its *rpoZ*-deleted mutant JW3624 under the standard procedure (78, 79). The ratio of the binding level of *rpoZ* mutant to that of wild type was plotted along the *E. coli* K-12 genome. The peaks located within open reading frames are shown in orange, while the peaks located inside the spacer are shown in green. The list of genes with high-level distribution of RpoZ-lacking RNAP is given in Table 1, while the locations of these genes along prophages are shown in Fig. 2.

The relative distributions between wild-type and RpoZ-defective mutant RNAP were similar along the entire *E. coli* K-12 genome (Fig. 1). One surprising finding is that the RpoZ-defective RNAP showed a high level of distribution, mostly within open reading frames (Fig. 1, orange background). Furthermore, these peaks of the high-level distribution of RpoZ-defective RNAP are located inside some prophages in the *E. coli* K-12 genome (see blue marks in Fig. 1 for the location of prophages). *E. coli* K-12 contains a total of 10 cryptic prophages (31, 32), i.e., CP4-6, DLP-12 (or Qsr), e14, Rac, Qin (or Kim), CP4-44, CPS-53 (or KpLE1), CPZ-55, CP4-57, and KpLE2, in this order along the *E. coli* K-12 genome and one short prophage segment, PR-X, on the genetic map (Table S1), altogether comprising about 3.6% of its genome sequence (33, 34). The prophage set is different between *E. coli* strains. For instance, prophages CP4-6, e14, Rac, Qin, CPS-53, and CP4-57 exist in both *E. coli* K-12 and pathogenic *E. coli* O157 strains, but DLP-12, CP4-44, PR-X, and CPZ-55 are present only in *E. coli* K-12 strains (32). On the other hand, *E. coli* O157 strains carry various types of O157-specific prophages. Each prophage of *E. coli* K-12 carries 9 (CPZ-55) to 43 (CP4-6) genes, but the functions are not known for most of these genes. The gene functions of prophages have been predicted based on the sequence similarity of the related original phages (for details, see Discussion).

10.1128/mSystems.00172-17.5 TABLE S1  List of prophages in *Escherichia coli* K-12. Download TABLE S1, PDF file, 0.04 MB. Copyright © 2018 Yamamoto et al. 2018 Yamamoto et al. This content is distributed under the terms of the Creative Commons Attribution 4.0 International license.

### Altered distribution of the RpoZ-defective RNAP inside specific genes.

**FIG 2:**
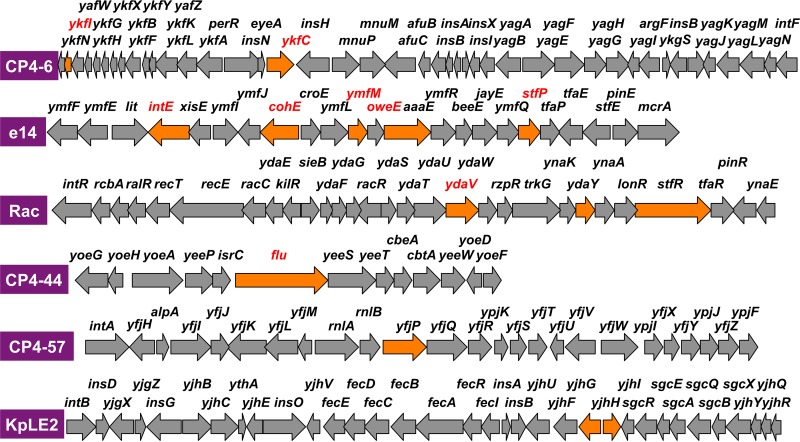
Locations of genes that exhibited increased levels of distribution of the RpoZ-defective RNAP. The genes that showed increased-level distribution of RpoZ-defective RNAP were identified by ChIP-chip analysis within six cryptic prophages of *E. coli* K-12 (Fig. 1; top 30 genes are in Table 1). Locations of these genes within each prophage are shown by orange arrows along the genetic map of the respective prophage. High-level occupancy was detected in particular within e14 and Rac prophages.

The ChIP-chip pattern indicates marked differences in the levels of RNAP occupancy inside some specific genes, in particular within prophages, between wild-type and *rpoZ*-deleted mutant strains. The marked increase in the distribution of RpoZ-defective RNAP was observed for about 30 positions by setting a cutoff level of 5 (occupancy relative to that of wild-type RNAP) (Fig. 1). The genes that exhibited the high-level distribution of RpoZ-defective RNAP are located within some cryptic prophage regions. The test DNA obtained by ChIP-chip screening often binds more than two probes of the tiling array, forming a single peak, and thus, the total amount of RNAP binding of each single peak was calculated by combining the fluorescence intensity hybridized for each array probe within a single and the same peak. Among the total of 30 peaks of high-level distribution of RpoZ-defective RNAP detected at the cutoff level of 5.0, the highest-level distribution of RpoZ-defective RNAP was identified inside the ORF of the CP4-44 prophage *flu* gene encoding the Ag43 autotransporter (Fig. 1). The relative binding level of RpoZ-defective RNAP for this site was 36.6 compared with wild-type RNAP (see Table S2 for details). Almost half (47%) of the high-level distribution of the mutant RNAP belonged to the genes that are organized in specific prophages, including CP4-6, e14, Rac, CP4-44, CP4-57, and KpLE2 (Fig. 1 shows the location of these prophages; for details, see Table S1). The increased distribution of RpoZ-defective RNAP was detected in multiple genes for e14 (5 genes), Rac (3 genes), CP4-6 (2 genes), and KpLE2 (2 genes) (Fig. 2). The set of genes that showed high-level distribution of RpoZ-lacking RNAP included *ymfN* (putative transcription factor [TF]), *ymfK* (putative repressor), *ycfK* (unknown protein), *intE* (predicted integrase), and *ymfM* (unknown protein) in e14 prophage; *ydaY* (pseudogene), *stfR* (predicted tail fiber protein), and *ydaV* (putative replication protein) in Rac prophage; *ykfC* (conserved protein) and *ykfI* (YkfI-YafW T-AT toxin) in CP4-6 prophage; and *yjhH* (predicted lyase) and *yjhG* (d-xylonate dehydrogenase) in KpLE2 prophage (Fig. 2 shows the locations of these genes in each prophage). Even though little is known about the functions of prophage-encoded proteins, these cryptic phages contribute to cell physiology such as cell growth, resistance to antibiotics, stress responses, and biofilm formation (34), implying that at least some of the prophage-encoded proteins are expressed in *E. coli* supposedly under specific conditions. The genes showing the high-level distribution of RpoZ-lacking RNAP are not clustered but scattered along each prophage (Fig. 2). Including these genes, transcription organization of the prophage genes is not known yet (for details, see Discussion).

10.1128/mSystems.00172-17.6 TABLE S2  Increased level of distribution of the *rpoZ* mutant RNAP in growing cells of *E. coli* K-12. Download TABLE S2, PDF file, 0.1 MB. Copyright © 2018 Yamamoto et al. 2018 Yamamoto et al. This content is distributed under the terms of the Creative Commons Attribution 4.0 International license.

Among the top 30 genes that exhibited increased distribution of the RpoZ-deleted RNAP, about half are carried by the host *E. coli* K-12 genome (Table 1) and are located within a group of transporter genes such as the genes encoding LsrAC transporter for cell-cell communication signal AI-2 and ModABC transporter for molybdate. Signal transduction apparatus is also included, such as RcsBC two-component system (TCS) phosphotransferase and NtrBC TCS sensor. It is noteworthy that the cell surface receptor for phage N4 is also included in this group. Some of these genes could be considered the research targets for detailed analysis of the role of RpoZ in the functional control of RNAP.

**TABLE 1:** Genes that showed increased binding of RpoZ-defective RNAP (ChIP-chip assay)^a^

Gene	Level	Function	Prophage
*flu*	82.5	Ag43 autotransporter	CP4-44
*lsrA*	45.4	Predicted AI-2 ABC transporter	
*ymfN*	42.8	Putative TF	e14
*ymfK*	32.2	Putative repressor	e14
*lsrC*	31.6	Predicted AI-2 ABC transporter	
*nfrA*	31.3	OM N4 receptor	
*paaE*	30.6	Predicted phenylacetyl-CoA epoxidase	
*ydbA*	29.8	Putative OM protein	
*ykfC*	22.8	Conserved protein	CP4-6
*ykfI*	21.9	YfkI-YafW T-AT toxin	CP4-6
*ybcH*	21.7	Hypothetical protein	
*paaF*	21.6	Putative dehydroadipyl-CoA hydratase	
*modC*	18.4	Molybdate ABC transporter	
*rcsD*	17.6	RcsBC TCS phosphotransfer intermediate	
*yjhH*	16.3	Predicted lyase/synthase	KpLE2
*galT*	16.2	Galactose-1-phosphate uridylyltransferase	
*ydaY*	15.8	Pseudogene	Rac
*stfR*	15.1	Predicted tail fiber protein	Rac
*modF*	15.1	Putative ABC transporter	
*ybhJ*	14.6	Putative hydratase	
*ycfK*	13.4	Putative protein	e14
*ydaV*	12.9	Putative DNA replication protein	Rac
*intE*	12.1	Putative integrase	e14
*ymfM*	12.0	Putative protein	e14
*yjhG*	11.6	d-Xylonate dehydrogenase	KpLE2
*yfjP*	11.2	Predicted GTP-binding protein	CP4-57
*nfrB*	11.0	NtrBC TCS sensor kinase	
*modA*	11.0	Molybdate ABC transporter	
*yhcD*	11.0	Putative fimbrial usher protein	
*ydbA*	10.9	Putative protein	

^a^ ChIP-chip assay was performed for *E. coli* K-12 wild type and its *rpoZ*-defective mutant. After tiling array analysis, the levels of RNAP binding were compared between two strains. The ratio between the binding of RpoZ-defective RNAP and that of wild-type RNAP was calculated for the entire probe along the genome. These values are listed in decreasing order in the Level column. When the gene is located inside prophages, the name of the prophage is given. OM, outer membrane; CoA, coenzyme A.

**FIG 3:**
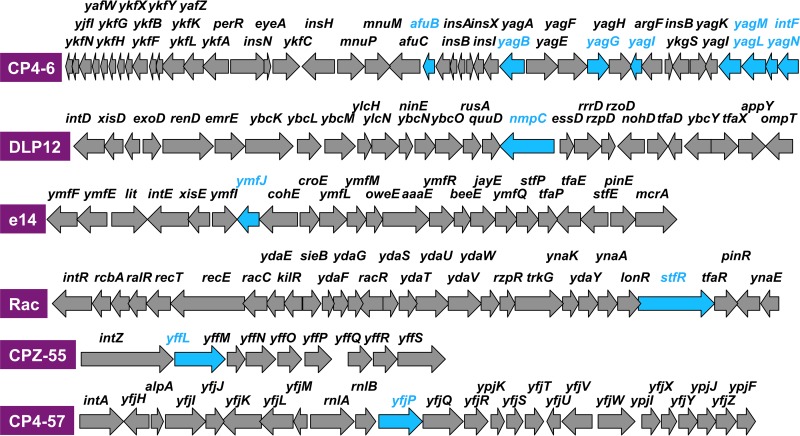
Locations of genes that exhibited decreased levels of distribution of the RpoZ-defective RNAP. The genes that showed decreased-level distribution of RpoZ-defective RNAP were identified by ChIP-chip analysis within six cryptic prophages of *E. coli* K-12 (Fig. 1; top 31 genes are in Table 3). Locations of these genes within each prophage are shown by blue arrows along the genetic map of the respective prophage. Decreased-level occupancy was detected in particular within CP4-6 prophage.

The ChIP-chip pattern also indicated decreased distribution of the RpoZ-deleted RNAP inside the ORF of specific genes, again mostly within some prophages (Table S3). Marked reduction in the distribution of mutant RNAP was observed for prophage CP4-6 (Fig. 3). The lowest difference, of 0.03, was observed inside the ORF of the CP4-6 prophage *yagN* and CPZ-55 prophage *yffN* genes (Table S3). Noteworthy is that the genes exhibiting the decreased distribution of the RpoZ-defective RNAP in the prophages are located within the so-called promoter islands containing promoter-like sequences (35). Although the level of constitutive promoters recognized by RpoD holoenzyme alone in the absence of additional supporting factors is low in the prophage regions (see below), the level of promoter islands is high in some prophage regions (35). One possibility is that these promoter-like sequences within the promoter islands might be differentially recognized by RNAP with and without RpoZ subunit (see below).

10.1128/mSystems.00172-17.7 TABLE S3  Decreased level of distribution of the *rpoZ* mutant RNAP in growing cells of *E. coli* K-12. Download TABLE S3, PDF file, 0.1 MB. Copyright © 2018 Yamamoto et al. 2018 Yamamoto et al. This content is distributed under the terms of the Creative Commons Attribution 4.0 International license.

### Transcription of the genes showing altered distribution of the RpoZ-defective RNAP.

The altered distribution of the RpoZ-defective RNAP in some specific genes might be correlated with altered transcription, pausing, and/or attenuation-termination. To search for possible relationships between altered distribution of RpoZ-defective RNAP and altered transcription, in particular within prophages, attempts were then made to directly measure transcripts from the genes that showed altered distribution of RpoZ-defective RNAP. From the set of genes that showed high-level distribution of RpoZ-defective RNAP (Table 1), we selected 17 genes, i.e., 5 from the prophages and 12 from the host genome (Table 2). Using reverse transcription-quantitative PCR (RT-qPCR), transcripts of these genes were measured for these genes. As expected, the *rpoZ* mRNA was not detectable in the *rpoZ-*defective mutant strain. Among the total of 17 genes examined, the level of transcript was more than 4-fold higher for 7 genes (*ydbA*, *ybdJ*, *yfiQ* [CP4-57], *paaE*, *ydaY* [Rec], *ybcH*, and *yhcD*) and the transcript level was more than 2-fold higher for 6 genes (*ymfN* [e14], *basS*, *yaiP*, *ybhA*, *ycfK*, and *ymfK* [e14]) (Table 3). This finding indicates that the high-level distribution of RpoZ-defective RNAP correlates with their high-level transcription.

**TABLE 2:** Transcription of genes that showed increased distribution of RpoZ-defective RNAP in prophages (qRT-PCR assay)^b^

Gene	Ratio^a^	Function	Prophage
*ydbA*	7.66 ± 6.24	Predicted autotransporter	
*ybhJ*	5.96 ± 1.93	Predicted hydratase	
*yfjQ*	4.79 ± 1.44	OM protein assembly complex	CP4-57
*paaE*	4.69 ± 2.16	1,2-Phenylacetyl-CoA epoxidase	
*ydaY*	4.69 ± 0.91	Phage protein	Rac
*ybcH*	4.54 ± 0.21	Predicted protein	
*yhcD*	4.53 ± 0.46	Predicted OM usher porin protein	
*ymfN*	3.42 ± 0.50	DNA-binding transcription regulator	e14
*basS*	3.27 ± 0.42	BasST TCS histidine kinase	
*yaiP*	2.87 ± 1.18	Predicted glucosyltransferase	
*ybhA*	2.32 ± 0.07	Pyridoxal phosphate/fructose-2P phosphatase	
*ycfK*	2.29 ± 1.31	Predicted protein (e14 prophage)	
*ymfK*	2.05 ± 0.08	DNA-binding transcription regulator	e14
*nfrB*	1.81 ± 0.35	NtrBC TCS histidine kinase (N4 receptor)	
*flu*	1.74 ± 0.60	Ag43 autotransporter	CP4-44
*isrC*	1.68 ± 0.49	IsrC sRNA (*flu* gene attenuation)	
*rcsD*	1.07 ± 0.19	RcsCDB phosphorelay phosphotransferase	

^a^ Ratio indicates the relative level of mRNA (*rpoZ* mutant/wild type). When the target gene is located inside a prophage, the name of the prophage is given.

^b^ The level of mRNA was determined for *E. coli* K-12 mutants lacking the *rpoZ* gene by using the qRT-PCR method. The genes were selected from the list showing the decreased distribution of RpoZ-defective RNAP (Table 1 and Fig. 3).

**TABLE 3:** Transcription of genes that showed decreased binding of RpoZ-defective RNAP inside prophages (ChIP-chip assay)^a^

Gene	Level	Function	Prophage
*yagM*	0.012	Putative protein	CP4-6
*yffL*	0.020	Putative protein	CPZ-55
*yagL*	0.023	DNA-binding protein	CP4-6
*yagB*	0.022	Predicted protein	CP4-6
*yagG*	0.026	Xyloside transporter	CP4-6
*wzxB*	0.029	O-antigen flippase	
*afuB*	0.030	Predicted ferric transporter	CP4-6
*intF*	0.032	Putative phage integrase	CP4-6
*wbbI*	0.036	Putative GalF transferase	
*yagN*	0.041	Putative protein	CP4-6
*stfR*	0.044	Putative membrane protein	Rac
*yehB*	0.047	Putative OM fimbrial usher protein	
*yjjJ*	0.053	Toxin-like protein	
*yagI*	0.060	XynR TF	CP4-6
*wbbH*	0.061	Putative O-antigen polymerase	
*napA*	0.065	Periplasmic nitrate reductase	
*ygeM*	0.067	Putative protein	
*waaI*	0.069	UDP-glucose:LPS glucosyltransferase	
*ymfJ*	0.072	Putative protein	e14
*nmpC*	0.075	Predicted OM porin NmpC	DLP
*frvA*	0.077	Fructose PTS enzyme IIA	
*yjiT*	0.078	Conserved protein	
*yqiK*	0.082	Putative protein	
*zraS*	0.083	Zinc-sensing ZraSR TCS sensor kinase	
*ypjC*	0.085	Pseudogene	
*yddG*	0.088	Aromatic amino acid exporter	
*yjgL*	0.094	Putative OM protein	
*yfjP*	0.095	Predicted GTP-binding protein	CP4-57
*waaP*	0.095	LP core heptose kinase	
*yciC*	0.098	Putative IM protein	
*chiA*	0.100	Endochitinase	

^a^ ChIP-chip assay was performed for *E. coli* K-12 wild type and its *rpoZ*-defective mutant. After tiling array analysis, the levels of RNAP binding were compared between two strains. The ratio of binding levels between the RpoZ-defective RNAP and wild-type RNAP was calculated for the entire probe along the genome. These values are listed in increasing order in the Level column. When the gene is located inside prophages, the name of the prophage is given. LPS, lipopolysaccharide; PTS, phosphotransferase; LP, lipoprotein; IM, inner membrane.

In order to identify possible influence of the decreased binding of RpoZ-defective RNAP on transcription, we then analyzed the levels of transcripts for seven representative genes (Table 4) from the list of decreased binding of the mutant RNAP (Table 3). After RT-qPCR, transcripts were found to markedly decrease for *yagM*, *yffL*, *yagN*, *yjfJ*, *yagL*, and *intF* (Table 4). This finding indicates that the decreased distribution of RpoZ-defective RNAP correlates, to a certain extent, with their decreased transcription. Taken together, we predicted that the altered distribution of RpoZ-defective RNAP reflects the altered transcription of particular genes by the RpoZ-defective RNAP. Possible mechanisms of how only a specific set of genes is differently transcribed by the RpoZ-defective RNAP remain to be solved in future. In this aspect, the identification of contact partners, which collaborate with RNAP in different manners in the presence and absence of RpoZ, could be important.

**TABLE 4:** Transcription of genes that showed decreased distribution of RpoZ-defective RNAP inside prophages (qRT-PCR assay)^b^

Designation	Gene	*rpoZ*/WT	Ratio^a^	Function	Prophage
1	*yagM*	10.29 ± 0.5	0.0972	Phage protein with unknown function	CP4-6
2	*yffL*	4.48 ± 0.5	0.2233	Phage protein with unknown function	CPZ-55
3	*yagN*	2.44 ± 0.6	0.4093	Phage protein with unknown function	CP4-6
4	*ymfJ*	1.62 ± 0.6	0.6184	Phage protein with unknown function	e14
5	*yagL*	1.54 ± 0.6	0.6405	Predicted transcription factor	CP4-6
6	*intF*	1.47 ± 0.5	0.6816	Putative phage integrase	CP4-6
7	*nmpC*	0.99 ± 0.9	1.0141	Putative OM protein	DPL
8	*afuB*	0.86 ± 0.6	1.1638	Predicted ferric transporter	CP4-6
a	*yjhH*	0.95 ± 0.7	1.0494	Putative lyase	KpLE2
b	*ykfC*	0.87 ± 0.5	1.1436	Phage protein with unknown function	CP4-6

^a^ Ratio indicates the relative level of mRNA (*rpoZ* mutant/wild type). When the target gene is located inside a prophage, the name of the prophage is given.

^b^ The level of mRNA was determined for *E. coli* K-12 mutants lacking the *rpoZ* gene by using the qRT-PCR method. The genes were selected from the list showing the decreased distribution of RpoZ-defective RNAP (Table 3 and Fig. 3). As references, two genes (a and b) were selected from the list of genes with increased distribution of RpoZ-defective RNAP (Table 2 and Fig. 2).

### Distribution of the constitutive promoters inside prophages.

The prophage genes are generally silent and are not expressed under the steady state of cell growth, but some genes are induced under specific conditions, leading to influence on cell growth, resistance to antibiotics, stress responses, and biofilm formation (for instance, see reference 34. For instance, the minor sigma factor FecI is encoded by the KpLE2 prophage and regulates only the divergently transcribed *fecABCDE* operon in the same prophage. This small regulon is employed by *E. coli* for utilization of the ferric citrate transport system (36).

**FIG 4:**
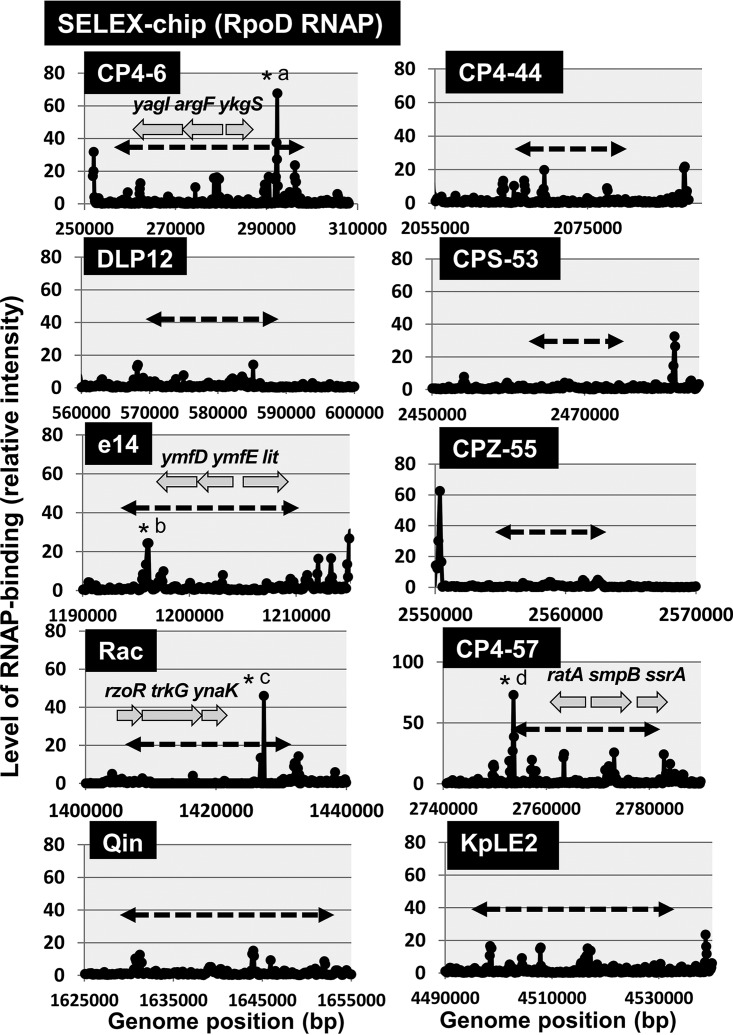
Locations of constitutive promoters of RNAP RpoD holoenzyme within prophages. Genomic SELEX screening was performed to search for promoters recognized by RNAP RpoD holoenzyme. Most of the prophages lack promoters with high-level activity. Significant peaks were detected in several prophages as indicated by asterisks: *a, *argF* promoter (CP4-6 prophage); *b, *lit* promoter (e14 prophage); *c, *trkG* promoter (Rac prophage); *d, *smpB* promoter (CP4-57 prophage boundary). See Fig. S2A for the binding sites of RNAP RpoD holoenzyme along the entire genome of *E. coli* K-12.

The promoters recognized by *E. coli* RNAP should be fewer in prophages, because in the case of *E. coli* phages, only early genes are transcribed by the host RNAP, but afterward, the modified host RNAP by phage gene products (in the case of T-even and lambdoid phages) or the phage-encoded RNAP (in the case of T7-type phages) is responsible for the transcription of late genes (37). Except for a limited number of characterized genes such as the *fec* regulon in prophage KpLE2, however, almost nothing is known of the locations of promoters inside the prophages. We then analyzed the distribution of promoters in each prophage using the genomic SELEX (systematic evolution of ligands by exponential enrichment) screening system. RNAP holoenzyme was reconstituted from the sigma-free core enzyme and 4-fold excess of purified RpoD sigma. The constitutive promoters were predicted based on the binding sites of this reconstituted RpoD holoenzyme alone in the absence of other DNA-binding proteins (38). A maximum total of 669 constitutive promoters were identified on the *E. coli* K-12 genome (38, 39) (see Fig. S2A for the distribution of RpoD constitutive promoters). Only a small number of the constitutive promoters were identified on prophages CP4-6, e14, and Rac (Fig. 4), supposedly each contributing to the transcription of the *argF* gene encoding ornithine carbamoyltransferase (CP4-6), the *lit* gene encoding protease for cleavage of EF-Tu in collaboration with Gol protein (e14), and the *trkG* gene encoding K^+^ transporter (Rac), respectively. *E. coli* K-12 contains two genes, *argF* and *argI*, both encoding the ornithine carbamoyltransferase involved in the synthesis of l-citrulline from carbamoyl phosphate and l-ornithine along the pathway of arginine biosynthesis. Thus, the *argF* gene is considered to be integrated into the prophage CP4-6, together with its promoter, after duplication or transposition of the original *argI* gene (40). Likewise, the *trkG* gene encoding a K^+^ transporter is closely related to the *trkH* gene in the genome of *E. coli* K-12 (41). Both TrkG and TrkH are active as low-affinity transporters of K^+^ and function in conjunction with TrkA, a membrane binding protein. Thus, the *trkG* gene must have been inserted, together with its promoter, into the prophage Rac. The *lit* gene product blocks late gene expression of phage T4, leading to phage exclusion (42). This inhibitory activity depends on Gol protein of T4 gene 23, together functioning as peptidase for cleavage of EF-Tu (43). The putative promoter of the *lit* gene is, however, activated only after mutation (42). Taken together, it is unlikely that prophages contain many promoters recognized by *E. coli* K-12 RNAP.

10.1128/mSystems.00172-17.2 FIG S2  (A) Location of the constitutive promoters of RNAP RpoD holoenzyme along the entire genome of *E. coli* K-12. Genomic SELEX screening was performed to search for the promoters recognized by RNAP RpoD holoenzyme as described in Materials and Methods. (B) Location of the binding sites of H-NS silencer along the entire genome of *E. coli* K-12. Genomic SELEX screening was performed to search for H-NS binding sites as described in Materials and Methods. Download FIG S2, PDF file, 0.6 MB. Copyright © 2018 Yamamoto et al. 2018 Yamamoto et al. This content is distributed under the terms of the Creative Commons Attribution 4.0 International license.

### Distribution of H-NS silencer along the prophages.

**FIG 5:**
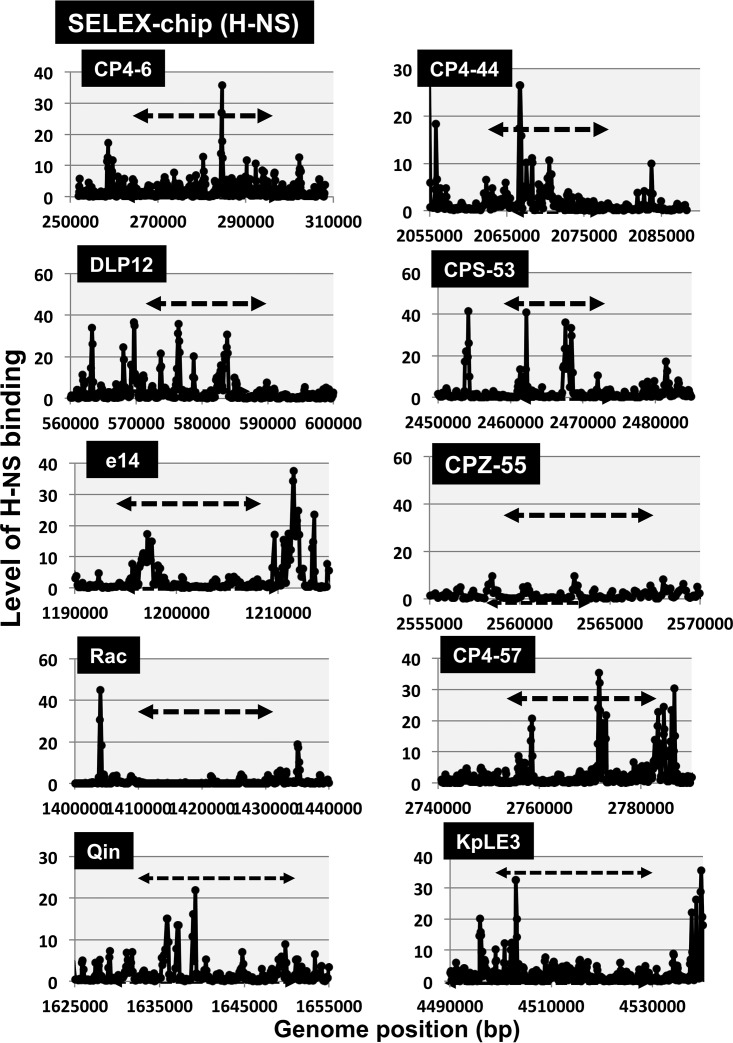
Locations of binding sites of H-NS silencer within prophages. Genomic SELEX screening was performed to search for the binding sites of H-NS within prophages. The H-NS binding sites thus detected are shown along each prophage region. See Fig. S2B for H-NS binding sites on the entire genome of *E. coli* K-12.

To avoid deleterious effects of the expression of foreign genes, including prophages, *E. coli* carries the gene silencing system, in which H-NS (44, 45) and Rho (46–48) are known to participate as sentries. Even though these two silencing players have apparently similar functions with respect to the gene silencing, the mechanism is different between H-NS and Rho. H-NS is one of the nucleoid-associated proteins with both an architectural role in genome folding and a global regulatory role of transcription (49). The altered transcription of a set of prophage genes by RpoZ-defective RNAP might be due to differences in the interaction of RpoZ-defective RNAP with prophage genome-associated H-NS silencer. We then analyzed the location of primary binding sites of H-NS along the genome of *E. coli* K-12 by using the Genomic SELEX screening system. A total of 987 binding sites were identified (38) (see Fig. S2B for the H-NS binding sites along the entire genome), of which a small number of H-NS binding sites were identified within specific prophages (Fig. 5). Noteworthy is that the strong binding sites of H-NS are almost absent for both e14 and Rac prophages, which showed the high-level distribution of RpoZ-defective RNAP. This finding implies that the binding of RpoZ-defective RNAP increased in the genes inside some prophages without strong binding sites for H-NS silencer.

### Altered phenotypes of the RNAP lacking RpoZ detected by PM analysis.

To see whether the differential occupancy of genes by RpoZ-less RNAP influences the phenotype of the cell, we studied the growth phenotype of the RpoZ-deleted strain with various carbon substrates and its tolerance to different environmental stresses like osmotic stress, pH, and antibiotic, using phenotype microarray (PM). PM technology allows us to find altered functions of genes by testing mutants for a large number of phenotypes simultaneously (50). An altered gene selection pattern of RpoZ-defective RNAP leads to the different phenotypes under various conditions tested. We then subjected both wild-type and *rpoZ*-defective *E. coli* strains to phenotype microarray (PM) assay.

**FIG 6:**
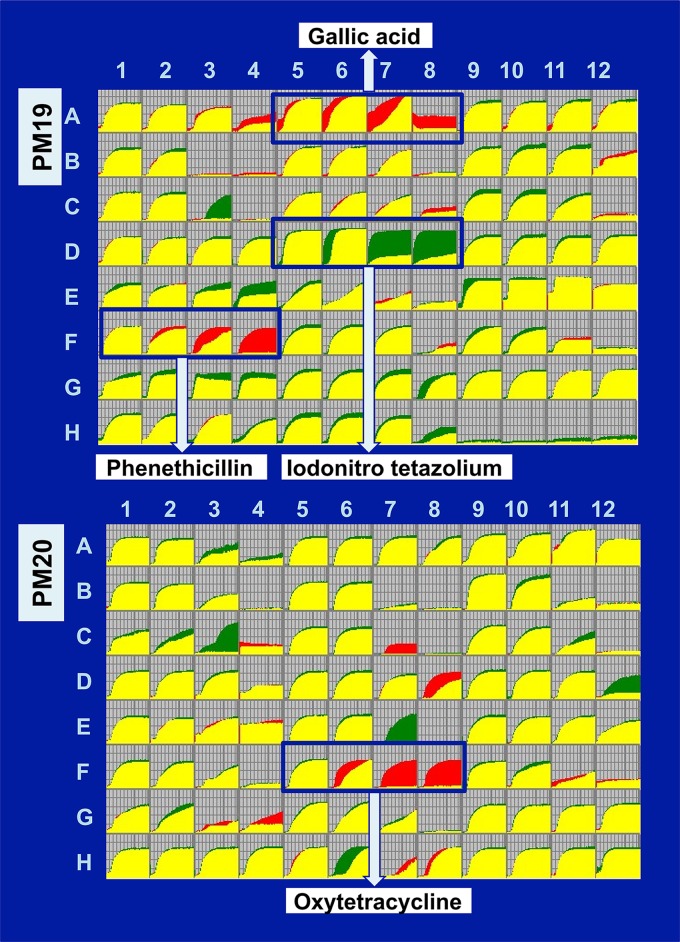
PM analysis of wild-type *E. coli* K-12 and its *rpoZ*-defective mutant. PM assay was performed for the combination of *E. coli* wild-type BW25113 and its *rpoZ*-deleted mutant JW3624 according to the standard procedure (86, 87). A typical pattern of the cell growth of the whole set of 20 PM plates is shown. The growth curve for wild type is shown in green, while that of the *rpoZ* mutant is shown in red. Differences in the growth rate were observed under several culture conditions as indicated.

The most prominent effect was seen on chemicals targeting the cell membrane, as the *rpoZ*-defective strain was remarkably sensitive toward sodium iodonitrotetrazolium (NT) violet (Fig. 6). NT violet is widely used for measurement of cellular redox activity or the respiratory activity in bacteria (50), indicating reduced transcription of the genes involved in respiratory metabolism for the *rpoZ* mutant. On the other hand, the growth of this mutant strain was significantly better than that of the wild type in the presence of gallic acid (PM19, A05-09) and phenethicillin (PM19, F01-04). Gallic acid, a type of phenolic acid, is known as a hydrated natural product of tannin and is commonly used in the pharmaceutical industry to produce a psychedelic alkaloid. Against bacteria, gallic acid is a toxic agent, a mutagen, and a modulator of amyloid formation (51, 52). These actions of gallic acid are considered to be attributable to changes in membrane properties such as charge and hydrophobicity. Phenethicillin is a semisynthetic acid-resistant penicillin, which is a methyl analog of phenoxymethyl penicillin. Penicillin group antibiotics bind to the penicillin-binding proteins (PBPs) and inhibit the cross-linking of peptidoglycan chains by PBPs (53), ultimately leading to weakening of the bacterial cell wall. Besides the antibiotics targeting the membrane, the *rpoZ*-defective mutant was sensitive to the ribosome-targeting oxytetracycline, a broad-spectrum tetracycline (PM20, F05-09) (Fig. 6). *E. coli* gains the resistance to tetracyclines through interference with its binding to ribosomes by ribosome protection proteins such as TetM (54). Resistance to tetracyclines is also mediated through decreased import or enhanced export of the drugs through membranes. In fact, the resistance by R factors is mediated through decrease of the intracellular level of tetracyclines (55).

The observed resistance of the *rpoZ*-defective mutant to these antibiotics detected by PM assay was then confirmed using individual liquid cultures in the presence of different concentrations of tetracycline (Fig. S3A) or penicillin (Fig. S3B). In the absence of drugs, the growth was retarded for the *rpoZ* mutant after late stationary phase. In the presence of these drugs, however, the growth retardation apparently disappeared. After prolonged culture, the growth of the *rpoZ* mutant was comparable to that of the wild type (Fig. S3) and continued longer than that of the wild type as observed by the PM assay (Fig. 6) and the tetrazolium reduction assay (Fig. S4). These observations altogether imply that the phenotypic resistance of the *rpoZ* mutant to these antibiotics could be attributable to the physiological modulation through some metabolic shifts due to altered transcription of as-yet-unspecified genes by the mutant RNAP. A close correlation has been established between the antibiotic sensitivity and the bacterial metabolism (56). Under starvation conditions after prolonged culture, the susceptibility to antibiotics decreases, leading to display of the phenotypic resistance. The altered distribution of RpoZ-defective RNAP in prophage regions and the altered sensitivity of the *rpoZ*-defective mutant to antibiotics both agree with the finding that the cryptic prophages influence the sensitivity to a variety of environments, including the sensitivity to antibiotics (34).

10.1128/mSystems.00172-17.3 FIG S3  (A) Influence of tetracycline on the growth of *E. coli* K-12 wild-type BW25112 and the *rpoZ*-deleted mutant JW3624. Cells were grown in LB medium in the absence or presence of the indicated concentrations of tetracycline for 24 h. (B) Influence of penicillin on the growth of *E. coli* K-12 wild-type BW25112 and the *rpoZ*-deleted mutant JW3624. Cells were grown in LB medium in the absence or presence of the indicated concentrations of penicillin for 24 h. Download FIG S3, PDF file, 1 MB. Copyright © 2018 Yamamoto et al. 2018 Yamamoto et al. This content is distributed under the terms of the Creative Commons Attribution 4.0 International license.

10.1128/mSystems.00172-17.4 FIG S4  (A) Influence of tetracycline on the metabolism of *E. coli* K-12 wild-type BW25112 and the *rpoZ*-deleted mutant JW3624. Cells were grown in LB medium in the absence or presence of the indicated concentrations of tetracycline. After 72 h, the metabolism was estimated by measuring the reduction of tetrazolium. (B) Influence of penicillin on the metabolism of *E. coli* K-12 wild-type BW25112 and the *rpoZ*-deleted mutant JW3624. Cells were grown in LB medium in the absence or presence of the indicated concentrations of penicillin. After 72 h, the metabolism was estimated by measuring the reduction of tetrazolium. Download FIG S4, PDF file, 0.04 MB. Copyright © 2018 Yamamoto et al. 2018 Yamamoto et al. This content is distributed under the terms of the Creative Commons Attribution 4.0 International license.

## DISCUSSION

*E. coli* RNAP functionally differentiates through two steps of protein-protein interaction: first, seven species of the sigma subunit, and second, more than 300 species of transcription factors (TFs) (57). TFs interact with one of the RNAP subunits for function (58–60). As a subunit of RNAP core enzyme, RpoZ might be involved in interaction with TFs. We then attempted to look into a broader perspective of the regulatory role played by RpoZ. Here, we identified the distribution of RNAP with and without RpoZ across the *E. coli* genome through ChIP-chip assay and found that the RNAP lacking RpoZ showed altered distribution within the prophage regions along the *E. coli* genome, supporting the prediction that RpoZ is involved in control of the regulatory function of RNAP.

A functional link between the RpoZ subunit and the stringent control has been suggested through its direct interaction with a nucleotide effector, ppGpp (17, 18, 24). Genetic and cross-linking studies of *E. coli* RNAP indicated that the direct binding of ppGpp to RpoB affects the catalytic activity of RNA polymerization (20, 21), while the mutational studies of *E. coli* RNAP and the recent crystal structure of RNAP-ppGpp complex showed that ppGpp binds at the interface of RpoB, RpoC, and RpoZ subunit (23–25). Besides this ppGpp-binding site 1, ppGpp-binding site 2 was identified at an interface between RNAP and DksA (27). In the absence of RpoZ, marked alteration was not identified for the set of hitherto-identified genes under stringent control such as rRNA genes; the ppGpp-binding site 2 might play a major role in the stringent control.

Horizontal transfer of foreign genes is a major contributor to the evolution of prokaryotic genomes (61). The well-characterized model bacterium *E. coli* K-12 carries a set of 10 cryptic prophages and a short prophage segment (32, 61). Overall, a total of approximately 304 genes, including predicted pseudogenes, exist in these prophages, and based on the similarity to phage genes, the functions have been predicted, but without experimental confirmation, for some genes in each prophage (see Table S1 in the supplemental material). One critical problem is whether these horizontally transferred genes are expressed and what the roles of these genes are in prophage survival inside the host *E. coli* K-12. Defective prophages Rac (62), e14 (63), DLP-12 (64), and Qin (65) are believed to carry some functional genes. Some of the prophage genes are beneficial to *E. coli*, including the genes encoding toxins and antibiotic resistance components for survival under various stressful natural conditions and for stable persistence in host animals. Sometimes, however, prophages kill *E. coli* through their induction. For instance, Rac repressor and integrase retain functional activity as conjugational transfer induces gene expression from the prophage and causes excision (66). In agreement with the expression of some functional genes from the Rac prophage, Rac is lethal to the host when its genes are expressed, resulting in the inhibition of cell division (67).

To avoid deleterious effects of prophages, *E. coli* carries the gene silencing system, in which H-NS (44, 45, 68) and Rho (46, 47) are known to participate as sentries. H-NS is known as a nucleoid-associated global silencer to prevent transcription. The silencing function of H-NS is interfered with by global regulators such as LeuO (69). We predicted that the level of H-NS binding is related to the alteration of transcription in the presence and absence of RpoZ. On the other hand, Rho acts as a transcription terminator. The altered transcription of a set of prophage genes by RpoZ-defective RNAP might also be due to the difference in the interaction of RpoZ-defective RNAP with termination factor Rho. Direct interaction of RNAP with Rho or Rho-Nus protein complexes has been suggested (3, 70). Involvement of Rho in the maintenance of some prophages has been suggested because the absence of the *rho* gene induces excision of defective prophages (47).

In the total of approximately 304 prophage genes, the genes for one RNAP sigma factor (FecI) and 14 TFs exist (Table S1), of which most are considered to control expression of the prophage genes. We have determined that the total number of constitutive promoters that are recognized by RNAP RpoD holoenzyme alone in the absence of supporting TFs is 492 to 669 (38). Along this line, the numbers of constitutive promoters recognized by the minor sigma factors were estimated to be 129 to 179 (RpoS), 101 to 142 (RpoH), 34 to 42 (RpoF), and 77 to 106 (RpoE) (71). In contrast, FecI is unique because it regulates only the divergently transcribed *fecABCDE* operon that encodes the ferric citrate transport system, suggesting that the regulatory target is still fixed within the KpLE2 prophage (36).

Among the total of 14 TFs, the regulatory target and function have been experimentally examined only for CP4-57-encoded AlpA (*intA* regulator) (72), DPL-12-encoded AppY (acid phosphatase regulator) (73), and CP4-6-encoded XynR (xylonate catabolism regulator) (74). Regulatory targets of these TFs seem to be fixed to the genes inside prophages. For instance, AlpA of CP4-57 regulates only the *intA* gene for excision of the CP4-57 gene (72). The polysaccharide xylan, one representative renewable plant hemicellulose biopolymer, consists of d-xylose. The main pathway for utilization of d-xylose by *E. coli* K-12 depends on the *xylFGH* and *xylAB* operons. XylR is a TF that activates d-xylose import (*xylFGH*) and catabolism (*xylAB*) genes (75). The expression level of XylR is controlled by DicF small RNA (sRNA) encoded by Qin prophage (76). *E. coli* lacks the oxidation pathway of xylose, but once the oxidized product xylonate is provided by coexisting microorganisms in nature, *E. coli* is able to catabolize xylonate with the use of CP4-6-encoded YagEF enzymes. XynR (renamed YagI) on CP4-6 prophage regulates only the adjacent *yagEF* genes on the same CP4-6 prophage (74), indicating that XynR is a rare single-target TF and its regulatory target is still fixed on the CP4-6 prophage genes. Thus, *E. coli* gained the system for utilizing plant-derived xylose from the prophages. In utilization of plant-derived xylan, host TF (XylR) and prophage TFs (XynR and DicF) collaborate, thereby contributing to the stable maintenance of prophages CP4-6 and Qin. Likewise, DLP-12-encoded AppY is induced under anaerobic conditions and, in collaboration with ArcA, plays a role in induction of the expression of the hydrogenase 1 operon (*hyaABCDEF*) (77). ArcA is the response regulator of the quinone-dependent ArcAB two-component signal transduction system to respond to the change in respiratory conditions. Prophage-encoded AppY and host ArcA collaborate for antirepression against the repressor IscR, the iron-sulfur cluster [2Fe-2S] regulator. This is another example of the novel mode of prophage-host interaction, in which the prophage-encoded TF collaborates with the host TF so as to modulate the spectrum of regulation targets. Except for these three TFs, the regulatory function is not known for the other 13 prophage-encoded TFs.

We conclude that not only structurally but functionally as well, RpoZ is crucial for transcription as it guides the transcription machinery to express the genes necessary for viability under various environmental stresses. Our results indicate a future need to explore the role of RpoZ in alteration of the RNAP functions, including the interaction with as-yet-unidentified TFs, including H-NS and Rho.

## MATERIALS AND METHODS

### Bacterial strains.

*Escherichia coli* strains BW25113 (parent strain) and JW3624 lacking the *rpoZ* gene (30) were used for all experiments. The strains were cultured in M9 medium (Difco) containing 0.4% glucose at 37°C. Cell growth was monitored by measuring the optical density at 600 nm (OD_600_).

### ChIP-chip analysis.

The ChIP-chip assay was carried out as previously described (78, 79) with a few modifications. BW25113 and JW3624 strains were grown in M9 glucose medium at 37°C to an optical density at 600 nm (OD_600_) of 0.2 and then incubated in M9 medium containing formaldehyde (final concentration of 1%) for 30 min for cross-linking between proteins and genomic DNA. After termination of the cross-linking reaction by the addition of glycine, cells were collected, washed, and lysed by addition of lysozyme. The lysates were sonicated using a digital Sonifier (Branson) to fragment the genome. After centrifugation, the supernatant of the whole-cell extract was subjected to immunoprecipitation with the anti-RpoA antibody (Neoclone)-coated protein A-Dynal Dynabeads (Invitrogen). RNAP-conjugated DNAs in immunoprecipitation fractions were digested by pronase (Roche), and then the free DNA fragments were purified using a QIAquick PCR purification kit (Qiagen). Recovered DNA fragments were amplified by PCR using a pair of random primers. The amplified DNA fragments from the wild-type strain were labeled with Cy3, while another sample from the *rpoZ* mutant was labeled with Cy5. The labeled DNAs were mixed and hybridized to a 43,450-feature *E. coli* tiling array (Oxford Gene Technology) (80, 81). After hybridization of samples to the DNA tiling array, the Cy5/Cy3 ratio was measured and the peaks of scanned patterns were plotted against the positions of DNA probes along the *E. coli* W3110 genome.

### Genomic SELEX screening.

The genomic SELEX screening was carried out as previously described (82, 83). A mixture of DNA fragments of the *E. coli* K-12 W3110 genome was prepared after sonication of purified genome DNA and cloned into a multicopy plasmid, pBR322. In each SELEX screening, the DNA mixture was regenerated by PCR. For SELEX screening, 5 pmol of the mixture of DNA fragments and 10 pmol reconstituted RNAP RpoD holoenzyme or purified H-NS were mixed in a binding buffer (10 mM Tris-HCl, pH 7.8, at 4°C, 3 mM magnesium acetate, 150 mM NaCl, and 1.25 mg/ml bovine serum albumin). RNAP RpoD holoenzyme was prepared by mixing the purified sigma-free core enzyme and a 4-fold molar excess of the overexpressed and purified RpoD sigma (38) while H-NS was purified from overexpressed cells (84). The sequences of DNA fragments obtained by the genomic SELEX screening were identified by a SELEX-chip method as described previously (82, 83). SELEX-chip data were submitted to the transcription factor profiling of *Escherichia coli* (TEC) at the National Institute of Genetics, Mishima, Japan (https://shigen.nig.ac.jp/ecoli/tec/top/; accession code RpoZ_ChIP).

### RT-qPCR.

Total RNA was prepared from *E. coli* cell as previously described (85). *E. coli* was grown in M9 glucose medium to an OD_600_ of 0.2 at 37°C with shaking. Cells were harvested, and total RNA was prepared using hot phenol. The concentration of total RNA was determined by measuring the absorbance at 260 nm, and its purity was checked by agarose gel electrophoresis. Next, total RNAs were transcribed to cDNA with random primers using the Primer Script first-strand cDNA synthesis kit (TaKaRa Bio), and quantitative PCR (qPCR) was conducted using SYBR green PCR master mix (Applied Biosystems) as previously described (38, 79). The primers used are described in Table S3 in the supplemental material. The cDNA templates were serially diluted to 2-fold and used in the qPCR assays. The levels of the 16S rRNA gene were used for normalization of data. The relative expression levels were quantified using the threshold cycle method presented by PE Applied Biosystems (PerkinElmer).

### PM.

Phenotype microarray (PM), a high-throughput technology for simultaneous testing of a large number of cellular phenotypes, was employed according to the manufacturer’s instructions (BioLog) (86, 87). In this study, PM was used for screening of the phenotypic differences between the wild-type strain and the *rpoZ*-deleted mutant strain. Growth difference was monitored by measuring the color intensity of oxidation of tetrazolium violet by NADH.
