# Supplementary figures and images for: Altered Distribution of RNA Polymerase Lacking the Omega Subunit within the Prophages along the Escherichia coli K-12 Genome
Source: mSystems. 2018 Feb 13;3(1):e00172-17. doi: 10.1128/mSystems.00172-17 (PMC5811629; doi:10.1128/mSystems.00172-17)

## Western blot analysis

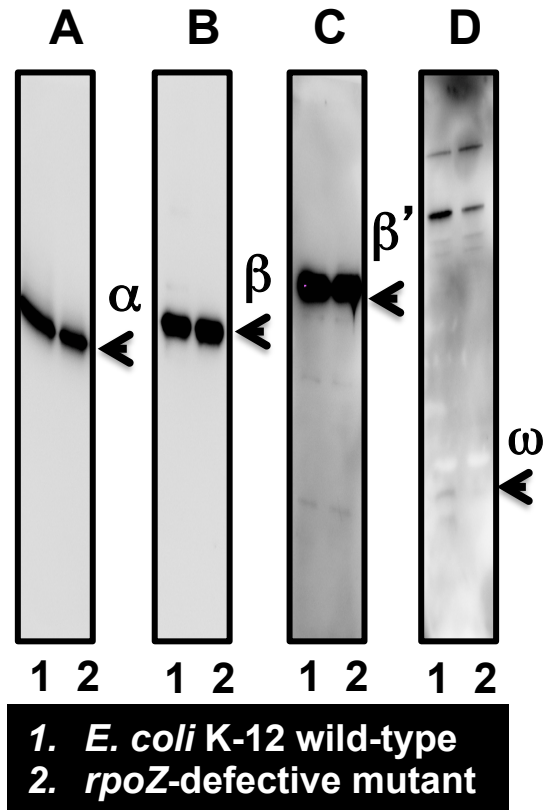

Yamamoto et al. (Fig. S1)

Supplement: FIG S1 [file sys001182181sf1.pdf]

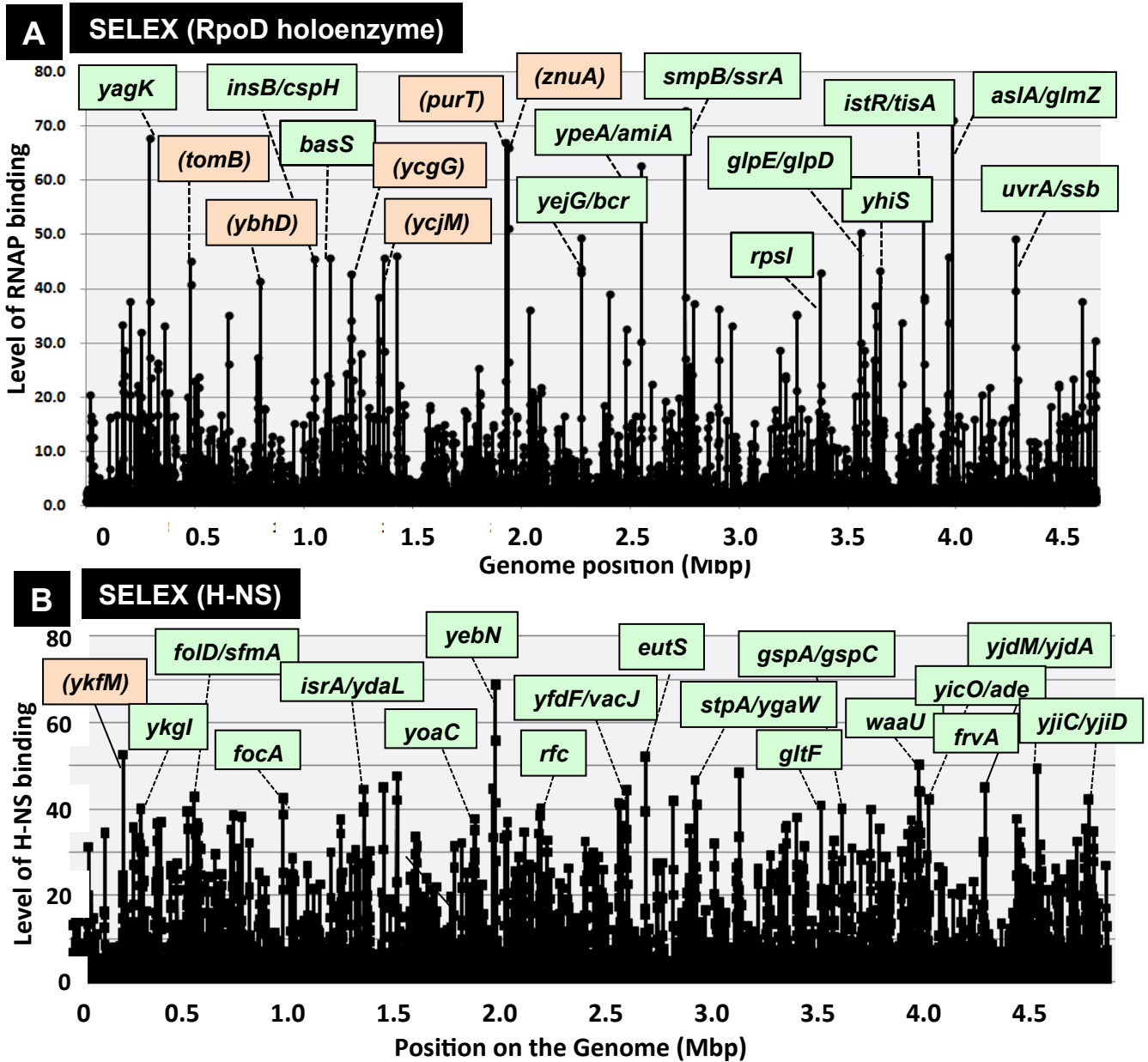

Yamamoto et al. (Fig. S2)

Supplement: FIG S2 [file sys001182181sf2.pdf]

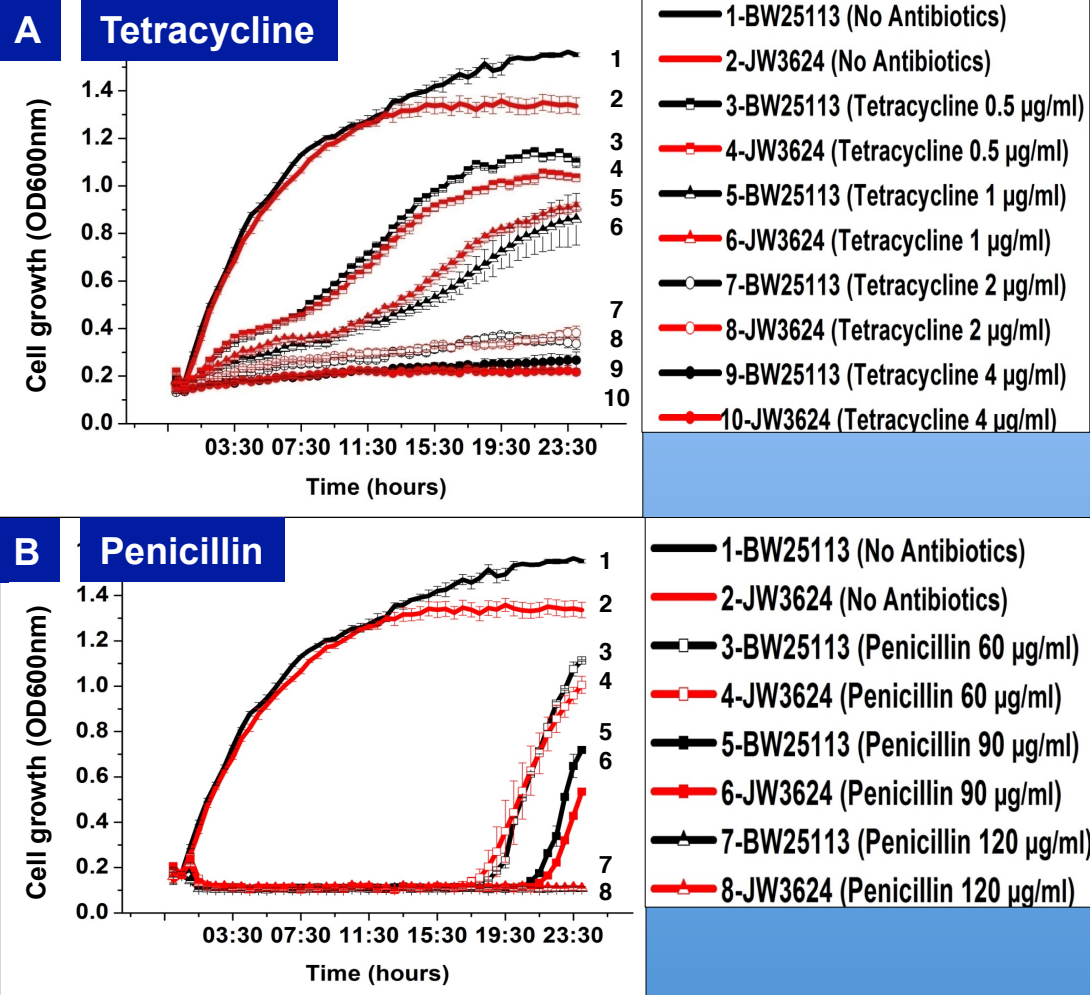

Yamamoto et al. (Fig. S3)

Supplement: FIG S3 [file sys001182181sf3.pdf]

Wild-type (BW25113) *rpoZ* mutant (JW3624)

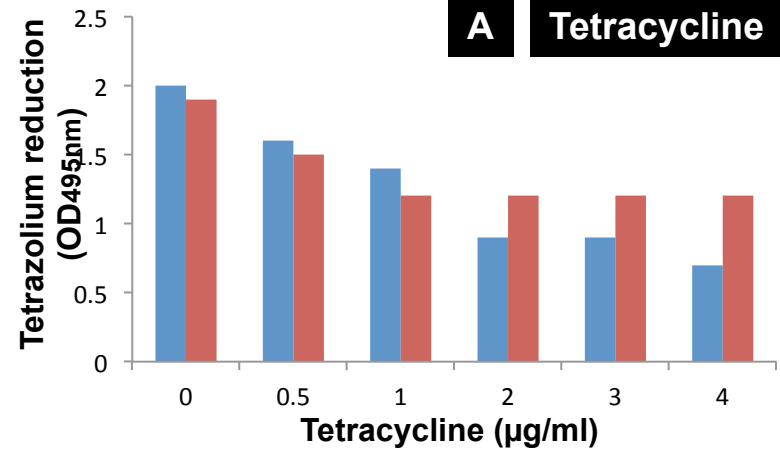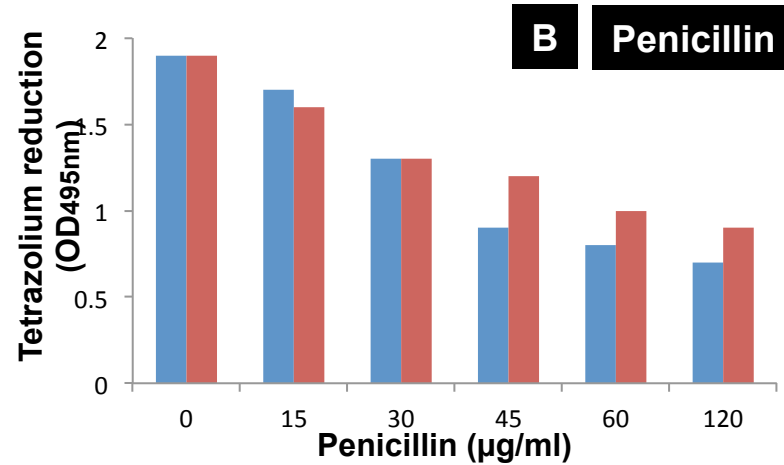

Supplement: FIG S4 [file sys001182181sf4.pdf]
