# Supplementary material for: Altered Distribution of RNA Polymerase Lacking the Omega Subunit within the Prophages along the Escherichia coli K-12 Genome
Source: mSystems. 2018 Feb 13;3(1):e00172-17. doi: 10.1128/mSystems.00172-17 (PMC5811629; doi:10.1128/mSystems.00172-17)
Supplement: TABLE S1 [file sys001182181st1.pdf]

Supplemental Table S1

List of Prophages in *Escherichia coli* K-12

1) Genes inside prophages

Prophage		Left end	Right end	Length	No. genes Total	Known	Unknown	Pseudo	TF	IS	Length w/o IS	Insertion site
**CP4-6**		262182	296489	34,308	48	17	26	5	5	8	27,332	*thrW*
**DLP12**	Qsr	564025	585326	21,302	36	23	11	2	2	2	18,849	*argU*
**e14**		1195443	1210635	15.193	22	13	8	1	2	0	15,193	*icd*
**Rac**		1409923	1432982	23,060	33	18	12	3	2	1	21,865	*ttcA*
**Qin**	Kim	1630310	1650767	20,458	40	25	13	2		1	19,752	*(ydfI-rspB)*
**CP4-44**		2065378	2077055	11,678	19	6	9	4		2	9,152	*(insH-yeeX)*
**PR-X**		2165222	2165850	629	2	1	1	0		0	629	*(cyaR-yegR)*
**CPS-53**	KpLE1	2464407	2474621	10,215	18	7	10	1		0	10,215	*argW*
**CPZ-55**	Eut	2556721	2563483	6,763	9	1	8	0		0	6,763	*(eutB-eutA)*
**CP4-57**		2753979	2776008	22,030	28	4	22	2	2	0	22,030	*(ssrA-ypjA)*
**KpLE2**		4494108	4534178	21,302	49	22	21	6	3		40,071	
				Total	304	137	141	26	16			

Note: The functions of prophages are mostly predicted based on the sequece similarity to the phage genes.

2) Regulator genes inside prophages

Siigma	Known (1)	**FecI**
TF	Known (4)	**AlpA, AppY, DicC, XynR**
TF	Unknown (10)	**PerR, RacR, SgcR, YagA, YbcM, YdaS, YfjR, YjhI, YmfN, YmfT**

Note: Regulatory functions have been experimentally identified only for FecI, AppY and XynR
