# Supplementary material for: Altered Distribution of RNA Polymerase Lacking the Omega Subunit within the Prophages along the Escherichia coli K-12 Genome
Source: mSystems. 2018 Feb 13;3(1):e00172-17. doi: 10.1128/mSystems.00172-17 (PMC5811629; doi:10.1128/mSystems.00172-17)
Supplement: TABLE S2 [file sys001182181st2.pdf]

Supplemental Table S2
Sites of Increased distribution of the *rpoZ* mutant RNAP

Position	Function	Left	D	RNAP site	D	Right	Function	WT	*rpoZ*
2070564	IsrC small RNA	*isrC*	>	*flu*	>	*yeeR*	CP4-44 prophage; predicted me	0.03	**36.61**
2072244	IsrC small RNA	*isrC*	>	*flu*	>	*yeeR*	CP4-44 prophage; predicted me	0.07	**13.88**
1454964	enylacetate degradation protein	*paaD*	>	*paaE*	>	*paaF*	predicted 2,3-dehydroadipyl-Co	0.08	**12.20**
1423932	ac prophage; conserved protein	*ynaK*	>	*ydaY*	>	*hantom Gene*		0.09	**10.88**
1465430	small protein	*ynbG*	<	*ydbA_1*	<	*insCD-2*		0.09	**10.68**
587048	EnvY transcriptional activator	*envY*	<	*ybcH*	<	*nfrA*	bacteriophage N4 receptor, out	0.09	**10.56**
1601540	LsrA	*lsrA*	>	*lsrC*	>	*lsrD*	LsrD	0.11	**9.45**
1196070	C0293 small RNA	*C0293*	>		<	*ymfD*	e14 prophage; predicted SAM-c	0.11	**9.44**
1204068	e14 prophage; predicted protein	*ymfM*	>	*ymfN*	>	*ymfR*	e14 prophage; predicted protei	0.11	**9.19**
1206850	14 prophage; conserved protein	*ymfQ*	>	*ycfK*	>	*ymfS*	e14 prophage; predicted protei	0.11	**9.16**
2312572	regulator of OmpF abundance	*micF*	>	*rcsD*	>	*rcsB*	RcsB	0.11	**9.11**
1201958	e14 prophage; predicted protein	*ymfJ*	<	*ymfK*	>	*ymfT*	e14 prophage; predicted DNA-b	0.11	**9.07**
262270	tRNA^{thrW}	*thrW*	>		<	*ykfI*	toxin of the YkfI-YafW toxin-anti	0.11	**8.98**
1203856	e14 prophage; predicted protein	*ymfM*	>	*ymfN*	>	*ymfR*	e14 prophage; predicted protei	0.12	**8.61**
1600072	LsrR transcriptional repressor	*lsrR*	<	*lsrA*	>	*lsrC*	LsrC	0.12	**8.47**
1465240	small protein	*ynbG*	<	*ydbA_1*	<	*insCD-2*		0.13	**7.83**
1203654	e14 prophage; predicted protein	*ymfM*	>	*ymfN*	>	*ymfR*	e14 prophage; predicted protei	0.13	**7.79**
589072	predicted protein	*ybcH*	<	*nfrA*	<	*nfrB*	bacteriophage N4 receptor, inne	0.14	**7.38**
1203134	binding transcriptional regulator	*ymfL*	>	*ymfM*	>	*ymfN*	e14 prophage; predicted DNA-b	0.14	**7.31**
1204672	e14 prophage; predicted protein	*ymfM*	>	*ymfN*	>	*ymfR*	e14 prophage; predicted protei	0.14	**7.24**
2877772	predicted integrase	*cas1*	<		<	*casE*	crRNA endonuclease	0.14	**7.18**
1201570	e14 prophage; predicted protein	*ymfJ*	<	*ymfK*	>	*ymfT*	e14 prophage; predicted DNA-b	0.14	**7.14**
587130	EnvY transcriptional activator	*envY*	<	*ybcH*	<	*nfrA*	bacteriophage N4 receptor, out	0.14	**7.09**
797342	ModC	*modC*	>	*ybhA*	>	*pgl*	6-phosphogluconolactonase	0.14	**7.06**
789534	galactokinase	*galK*	<	*galT*	<	*galE*	UDP-glucose 4-epimerase mon	0.14	**7.03**
3361232	periplasmic chaperone protein	*yhcA*	>	*yhcD*	>	*ychE_1*	predicted protein, N-ter fragmen	0.14	**7.00**
1202030	e14 prophage; predicted protein	*ymfJ*	<	*ymfK*	>	*ymfT*	e14 prophage; predicted DNA-b	0.14	**6.96**
4522660	element; predicted dehydratase	*yjhG*	<	*yjhH*	<	*yjhI*	KpLE2 phage-like element; pre	0.15	**6.88**
587256	predicted protein	*ybcH*	<	*nfrA*	<	*nfrB*	bacteriophage N4 receptor, inne	0.15	**6.88**
1425368	*Phantom Ger*		>	*ynaA*	>	*lomR_1*	Rac prophage; predicted protei	0.15	**6.82**
2071650	IsrC small RNA	*isrC*	>	*flu*	>	*yeeR*	CP4-44 prophage; predicted me	0.15	**6.76**
1197950	edicted inner membrane protein	*ymfE*	<	*lit*	<	*intE*	e14 prophage; predicted integra	0.15	**6.75**
1600368	LsrR transcriptional repressor	*lsrR*	<	*lsrA*	>	*lsrC*	LsrC	0.15	**6.69**
1429466	redicted protein, C-ter fragment	*lomR_2*	>	*stfR*	>	*tfaR*	Rac prophage; predicted tail fib	0.15	**6.65**
1601656	LsrA	*lsrA*	>	*lsrC*	>	*lsrD*	LsrD	0.15	**6.59**
1420040	ac prophage; conserved protein	*ydaU*	>	*ydaV*	>	*ydaW*	Rac prophage; predicted DNA-b	0.15	**6.58**
590934	ceptor, outer membrane protein	*nfrA*	<	*nfrB*	<	*cusS*	CusS	0.15	**6.55**
1209732	site-specific DNA recombinase	*pinE*	>	*mcrA*	>	*icdC*	conserved protein	0.16	**6.41**
4330870	ProP	*proP*	>	*basS*	<	*basR*	BasR transcriptional regulator	0.16	**6.41**
1601444	LsrA	*lsrA*	>	*lsrC*	>	*lsrD*	LsrD	0.16	**6.28**
792438	glucose 4-epimerase monomer	*galE*	<	*modF*	<	*modE*	ModE	0.16	**6.27**
1418472	binding transcriptional regulator	*racR*	<	*ydaS*	>	*ydaT*	Rac prophage; predicted protei	0.16	**6.26**
1730972	Rnt	*rnt*	>	*lhr*	<	*grxD*	glutaredoxin 4	0.16	**6.26**
1471542	transposase of IS30	*insI-2*	>	*ydbA_2*	>	*ydbC*	predicted oxidoreductase, NAD	0.16	**6.17**
1200944	e14 prophage; predicted protein	*ymfH*	<	*ymfI*	<	*ymfJ*	e14 prophage; predicted protei	0.16	**6.16**
4522072	YjhF Gnt tranporter	*yjhF*	<	*yjhG*	<	*yjhH*	predicted lyase/synthase	0.16	**6.12**
1600146	LsrR transcriptional repressor	*lsrR*	<	*lsrA*	>	*lsrC*	LsrC	0.16	**6.12**
2766158	-57 prophage; predicted protein	*yfjO*	>	*yfjP*	>	*yfjQ*	CP4-57 prophage; predicted pr	0.16	**6.10**
157546	ted fimbrial-like adhesin protein	*yadN*	<	*folK*	<	*pcnB*	poly(A) polymerase I	0.17	**6.05**
382950	predicted protein	*yaiF*	<	*yaiP*	<	*yaiS*	conserved protein	0.17	**6.03**
1455532	ylacetyl-CoA epoxidase subunit	*paaE*	>		>	*paaF*	predicted 2,3-dehydroadipyl-Co	0.17	**5.94**

2070054	IsrC small RNA	*isrC*	>	*flu*	>	*yeeR*	CP4-44 prophage; predicted me	0.17	**5.92**
1465342	small protein	*ynbG*	<	*ydbA_1*	<	*insCD-2*		0.17	**5.90**
1453732	ylacetyl-CoA epoxidase subunit	*paaB*	>	*paaC*	>	*paaD*	phenylacetate degradation prot	0.17	**5.78**
1599758	LsrR transcriptional repressor	*lsrR*	<	*lsrA*	>	*lsrC*	LsrC	0.17	**5.73**
1456072	ylacetyl-CoA epoxidase subunit	*paaE*	>	*paaF*	>	*paaG*	predicted ring 1,2-epoxyphenyla	0.17	**5.73**
2074950	e; predicted DNA repair protein	*yeeS*	>	*yeeT*	>	*yeeU*	CP4-44 prophage; antitoxin of t	0.18	**5.71**
4572470	conserved protein	*yjiT*	>	*yjiV*	<	*mcrC*	MrcC subunit of 5-methylcytosin	0.18	**5.63**
794844	e cell envelope stress response	*ybhT*	>	*modA*	>	*modB*	ModB	0.18	**5.61**
1456272	3-dehydroadipyl-CoA hydratase	*paaF*	>		>	*paaG*	predicted ring 1,2-epoxyphenyla	0.18	**5.60**
803372	YbhI DASS Transporter	*ybhI*	>	*ybhJ*	<	*ybhC*	outer membrane lipoprotein	0.18	**5.58**
1202338	ge; predicted repressor protein	*ymfK*	<	*ymfT*	>	*ymfL*	e14 prophage; predicted DNA-b	0.18	**5.57**
4522130	element; predicted dehydratase	*yjhG*	<		<	*yjhH*	predicted lyase/synthase	0.18	**5.54**
1205158	e14 prophage; predicted protein	*ymfR*	>	*ymfO*	>	*ymfP*	e14 prophage; conserved prote	0.18	**5.48**
2792144	ate aminotransferase monomer	*gabT*	>		>	*gabP*	GabP APC transporter	0.18	**5.46**
1455370	enylacetate degradation protein	*paaD*	>	*paaE*	>	*paaF*	predicted 2,3-dehydroadipyl-Co	0.18	**5.44**
1465542	small protein	*ynbG*	<	*ydbA_1*	<	*insCD-2*		0.18	**5.43**
383064	predicted protein	*yaiF*	<	*yaiP*	<	*yaiS*	conserved protein	0.18	**5.43**
762354	SucB	*sucB*	>	*sucC*	>	*sucD*	succinyl-CoA synthetase, &alph	0.18	**5.41**
1199130	phage exclusion; e14 prophage	*lit*	>	*intE*	<	*xisE*	e14 prophage; predicted excisio	0.19	**5.38**
269370	; predicted GTP-binding protein	*ykfA*	<	*perR*	>	*insN-1*	CP4-6 prophage; partial regulat	0.19	**5.33**
265342	4-6 prophage; predicted protein	*ykfB*	<		<	*yafY*	CP4-6 prophage; inner membra	0.19	**5.30**
795956	ModB	*modB*	>	*modC*	<	*ybhA*	pyridoxal phosphatase/fructose	0.19	**5.24**
1601048	LsrA	*lsrA*	>		>	*lsrC*	LsrC	0.19	**5.24**
263962	e; predicted DNA repair protein	*ykfG*	<		<	*yafX*	CP4-6 prophage; predicted prot	0.19	**5.23**
1456552	3-dehydroadipyl-CoA hydratase	*paaF*	>	*paaG*	>	*paaH*	3-hydroxyadipyl-CoA dehydroge	0.19	**5.22**
1455966	ylacetyl-CoA epoxidase subunit	*paaE*	>	*paaF*	>	*paaG*	predicted ring 1,2-epoxyphenyla	0.19	**5.19**
132632	putative membrane protein	*yacH*	<	*acnB*	>	*yacL*	conserved protein	0.19	**5.16**
2429734	cell division protein DedD	*dedD*	<	*folC*	<	*accD*	acetyl-CoA carboxyltransferase	0.19	**5.16**
1197150	M-dependent methyltransferase	*ymfD*	<	*ymfE*	>	*lit*	Lit, cell death peptidase; phage	0.20	**5.12**
2767172	; predicted GTP-binding protein	*yfjP*	>	*yfjQ*	>	*yfjR*	CP4-57 prophage; predicted DN	0.20	**5.10**
2069568	IsrC small RNA	*isrC*	>		>	*flu*	CP4-44 prophage; antigen 43 (A	0.20	**5.08**
271644	ed by insertion of IS30 element	*insO-1*	>		>	*ykfC*	CP4-6 prophage; conserved pro	0.20	**5.06**
797972	se/fructose 1,6-bisphosphatase	*ybhA*	<	*pgl*	<	*ybhD*	predicted DNA-binding transcrip	0.20	**5.06**
803452	YbhI DASS Transporter	*ybhI*	>	*ybhJ*	<	*ybhC*	outer membrane lipoprotein	0.20	**5.05**
790246	1-phosphate uridylyltransferase	*galT*	<		<	*galE*	UDP-glucose 4-epimerase mon	0.20	**5.04**
1201266	e14 prophage; predicted protein	*ymfI*	>	*ymfJ*	<	*ymfK*	e14 prophage; predicted repres	0.20	**5.03**
1461158	β-ketoadipyl-CoA thiolase	*paaJ*	>	*paaK*	>	*paaX*	PaaX	0.20	**5.00**
1203942	e14 prophage; predicted protein	*ymfM*	>	*ymfN*	>	*ymfR*	e14 prophage; predicted protein	0.20	**4.96**
1204338	e14 prophage; predicted protein	*ymfM*	>	*ymfN*	>	*ymfR*	e14 prophage; predicted protein	0.20	**4.96**
796262	ModB	*modB*	>	*modC*	<	*ybhA*	pyridoxal phosphatase/fructose	0.20	**4.94**
1205436	14 prophage; conserved protein	*ymfO*	>		>	*ymfP*	e14 prophage; conserved prote	0.20	**4.92**
272152	ed by insertion of IS30 element	*insO-1*	>	*ykfC*	<	*insH-1*	IS5 transposase and trans-activ	0.20	**4.92**
1423850	ac prophage; conserved protein	*ynaK*	>	*ydaY*	>	*hantom Gene*		0.20	**4.89**
1420636	ac prophage; conserved protein	*ydaU*	>	*ydaV*	>	*ydaW*	Rac prophage; predicted DNA-b	0.20	**4.88**
2995732		*insC-4*	<		<	*ygeP*	predicted protein	0.21	**4.84**
679334	served outer membrane protein	*ybeT*	<	*ybeU*	>	*djlC*	Hsc56, co-chaperone of Hsc62	0.21	**4.84**
382470	predicted protein	*yaiF*	<	*yaiP*	<	*yaiS*	conserved protein	0.21	**4.84**
2560934	-55 prophage; predicted protein	*yffO*	>	*yffP*	>	*yffQ*	CPZ-55 prophage; predicted pr	0.21	**4.82**
589344	predicted protein	*ybcH*	<	*nfrA*	<	*nfrB*	bacteriophage N4 receptor, inne	0.21	**4.78**
1455740	ylacetyl-CoA epoxidase subunit	*paaE*	>	*paaF*	>	*paaG*	predicted ring 1,2-epoxyphenyla	0.21	**4.77**
2767948	-57 prophage; predicted protein	*yfjQ*	>	*yfjR*	>	*ypjK*	CP4-57 prophage; predicted inn	0.21	**4.77**
262534	tRNA^{thrW}	*thrW*	>		<	*ykfI*	toxin of the YkfI-YafW toxin-anti	0.21	**4.75**
1202662	binding transcriptional regulator	*ymfT*	>	*ymfL*	>	*ymfM*	e14 prophage; predicted protein	0.21	**4.74**
383868	conserved protein	*yaiS*	<		>	*tauA*	TauA	0.21	**4.74**

1200254	rophage; predicted excisionase	*xisE*	<		<	*ymfH*	e14 prophage; predicted protei	0.21	**4.73**
1197732	edicted inner membrane protein	*ymfE*	<		>	*lit*	Lit, cell death peptidase; phage	0.21	**4.73**
1206454	14 prophage; conserved protein	*ymfP*	>	*ymfQ*	>	*ycfK*	e14 prophage; predicted protei	0.21	**4.73**
1471442	transposase of IS30	*insI-2*	>	*ydbA_2*	>	*ydbC*	predicted oxidoreductase, NAD	0.21	**4.72**
2768670	binding transcriptional regulator	*yfjR*	>	*ypjK*	>	*yfjS*	CP4-57 prophage; inner memb	0.21	**4.71**
1598340	autoinducer-2 kinase	*lsrK*	<		<	*lsrR*	LsrR transcriptional repressor	0.21	**4.70**
4522530	element; predicted dehydratase	*yjhG*	<	*yjhH*	<	*yjhI*	KpLE2 phage-like element; pre	0.21	**4.67**
4522266	element; predicted dehydratase	*yjhG*	<	*yjhH*	<	*yjhI*	KpLE2 phage-like element; pre	0.21	**4.67**
4569338	predicted transposase	*yjiQ*	>	*yjiR*	>	*yjiS*	conserved protein	0.22	**4.64**
2072072	IsrC small RNA	*isrC*	>	*flu*	>	*yeeR*	CP4-44 prophage; predicted m	0.22	**4.64**
1450164	TynA	*tynA*	<	*paaZ*	>	*paaA*	predicted ring 1,2-phenylacetyl-	0.22	**4.63**
2076030	eV-YeeU toxin-antitoxin system	*yeeV*	>	*yeeW*	>	*yoeF*	conserved protein	0.22	**4.59**
1454158	ylacetyl-CoA epoxidase subunit	*paaC*	>	*paaD*	>	*paaE*	predicted ring 1,2-phenylacetyl-	0.22	**4.59**
287630	putative β-xylosidase	*yagH*	>		<	*yagI*	CP4-6 prophage; predicted DN	0.22	**4.57**
268354	; predicted GTP-binding protein	*ykfA*	<		<	*perR*	PerR transcriptional regulator	0.22	**4.56**
1428734	redicted protein, C-ter fragment	*lomR_2*	>	*stfR*	>	*tfaR*	Rac prophage; predicted tail fib	0.22	**4.55**
792358	glucose 4-epimerase monomer	*galE*	<	*modF*	<	*modE*	ModE	0.22	**4.55**
800930	binding transcriptional regulator	*ybhD*	<	*ybhH*	>	*ybhI*	YbhI DASS Transporter	0.22	**4.54**
2473052	E1) prophage; predicted protein	*yfdQ*	>	*yfdR*	>	*yfdS*	CPS-53 (KpLE1) prophage; pre	0.22	**4.53**
272358	ed by insertion of IS30 element	*insO-1*	>	*ykfC*	<	*insH-1*	IS5 transposase and trans-acti	0.22	**4.50**
1458754	lrogenase (NAD⁺)	*paaH*	>	*paaI*	>	*paaJ*	β-ketoadipyl-CoA thiolase	0.22	**4.47**
592370	ceptor, outer membrane protein	*nfrA*	<	*nfrB*	<	*cusS*	CusS	0.22	**4.45**
4517736		*insB-7_2*	>	*yjhU*	<	*yjhF*	YjhF Gnt tranporter	0.23	**4.43**
1600932	LsrR transcriptional repressor	*lsrR*	<	*lsrA*	>	*lsrC*	LsrC	0.23	**4.43**
1454370	ylacetyl-CoA epoxidase subunit	*paaC*	>	*paaD*	>	*paaE*	predicted ring 1,2-phenylacetyl-	0.23	**4.42**
940230	seryl-tRNA synthetase	*serS*	>	*dmsA*	>	*dmsB*	dimethyl sulfoxide reductase, c	0.23	**4.40**
2312436	regulator of OmpF abundance	*micF*	>	*rcsD*	>	*rcsB*	RcsB	0.23	**4.39**
2848130	hydrogenase 3, Fe-S subunit	*hycB*	<	*hycA*	>	*hypA*	accessory protein for nickel inc	0.23	**4.39**
2070640	IsrC small RNA	*isrC*	>	*flu*	>	*yeeR*	CP4-44 prophage; predicted m	0.23	**4.38**
795332	ModA	*modA*	>	*modB*	>	*modC*	ModC	0.23	**4.36**
797654	se/fructose 1,6-bisphosphatase	*ybhA*	<		>	*pgl*	6-phosphogluconolactonase	0.23	**4.35**
793560	ModF	*modF*	<	*modE*	>	*ybhT*	small membrane protein involve	0.23	**4.34**
1196168	C0293 small RNA	*C0293*	>	*ymfD*	<	*ymfE*	e14 prophage; predicted inner	0.23	**4.33**
1459732	yphenylacetyl-CoA thioesterase	*paaI*	>	*paaJ*	>	*paaK*	phenylacetate-CoA ligase	0.23	**4.32**
2933440		*fucA*	<	*fucP*	>	*fucI*		0.23	**4.32**
2068966	hypothetical protein	*yoeE*	>	*yeeP*	>	*isrC*	IsrC small RNA	0.23	**4.30**
1209632	site-specific DNA recombinase	*pinE*	>	*mcrA*	>	*icdC*	conserved protein	0.23	**4.29**
272534	ed by insertion of IS30 element	*insO-1*	>	*ykfC*	<	*insH-1*	IS5 transposase and trans-acti	0.23	**4.27**
1200160	4 prophage; predicted integrase	*intE*	<	*xisE*	<	*ymfH*	e14 prophage; predicted protei	0.23	**4.27**
262764	tRNA^{thrW}	*thrW*	>	*ykfI*	<	*yafW*	antitoxin of the YkfI-YafW toxin-	0.23	**4.26**
2762334	57 prophage; conserved protein	*yfjK*	<	*yfjL*	<	*yfjM*	CP4-57 prophage; predicted pr	0.24	**4.24**
795838	ModB	*modB*	>	*modC*	<	*ybhA*	pyridoxal phosphatase/fructose	0.24	**4.24**
1428632	redicted protein, C-ter fragment	*lomR_2*	>	*stfR*	>	*tfaR*	Rac prophage; predicted tail fib	0.24	**4.23**
1598262	autoinducer-2 kinase	*lsrK*	<		<	*lsrR*	LsrR transcriptional repressor	0.24	**4.22**
3642234	M-dependent methyltransferase	*yhiQ*	<	*prlC*	>	*yhiR*	protein involved in utilization of	0.24	**4.21**
2072336	IsrC small RNA	*isrC*	>	*flu*	>	*yeeR*	CP4-44 prophage; predicted m	0.24	**4.21**
287038	YagG GPH Transporter	*yagG*	>	*yagH*	<	*yagI*	CP4-6 prophage; predicted DN	0.24	**4.20**
2592530	esterase	*ypfH*	<	*tmcA*	<	*ypfJ*	conserved protein	0.24	**4.20**
1206970	14 prophage; conserved protein	*ymfQ*	>	*ycfK*	>	*ymfS*	e14 prophage; predicted protei	0.24	**4.19**
789930	galactokinase	*galK*	<	*galT*	<	*galE*	UDP-glucose 4-epimerase mon	0.24	**4.18**
788562	galactose-1-epimerase	*galM*	<	*galK*	<	*galT*	galactose-1-phosphate uridylyl	0.24	**4.17**
1967156	CheB	*cheB*	<	*cheR*	<	*tap*	Tap	0.24	**4.16**
2522850	pB xanthosine MFS transporter	*xapB*	<	*xapA*	>	*yfeN*	conserved outer membrane pro	0.24	**4.15**
262434	tRNA^{thrW}	*thrW*	>		<	*ykfI*	toxin of the YkfI-YafW toxin-ant	0.24	**4.14**

1414360	recombinase, DNA renaturation	*recT*	<	*recE*	<	*racC*	Rac prophage; predicted protei	0.24	**4.13**
4569036	predicted transposase	*yjiQ*	>	*yjiR*	>	*yjiS*	conserved protein	0.24	**4.12**
1602248	LsrC	*lsrC*	>	*lsrD*	>	*lsrB*	LsrB	0.24	**4.12**
796568	ModB	*modB*	>	*modC*	<	*ybhA*	pyridoxal phosphatase/fructose	0.24	**4.10**
274956	transposase and trans-activator	*insH-1*	<	*mmuP*	>	*mmuM*	homocysteine methyltransferas	0.24	**4.10**
589844	predicted protein	*ybcH*	<	*nfrA*	<	*nfrB*	bacteriophage N4 receptor, inn	0.24	**4.10**
2312230	regulator of OmpF abundance	*micF*	>	*rcsD*	>	*rcsB*	RcsB	0.24	**4.10**
109870	n (General Secretory Pathway)	*secM*	>	*secA*	>	*mutT*	dGTP pyrophosphohydrolase	0.24	**4.08**
1453334	ylacetyl-CoA epoxidase subunit	*paaB*	>	*paaC*	>	*paaD*	phenylacetate degradation prot	0.25	**4.07**
2778358	YpjF-YfjZ toxin-antitoxin system	*ypjF*	>	*ypjA*	<	*pinH*	predicted invertase fragment	0.25	**4.07**
587436	predicted protein	*ybcH*	<	*nfrA*	<	*nfrB*	bacteriophage N4 receptor, inn	0.25	**4.07**
611372	hosphopantetheinyl transferase	*entD*	<	*fepA*	>	*fes*	enterochelin esterase	0.25	**4.05**
589634	predicted protein	*ybcH*	<	*nfrA*	<	*nfrB*	bacteriophage N4 receptor, inn	0.25	**4.04**
380330	acyl transferase (pseudogene)	*yaiX*	<		>	*insCD-1*	IS2 element transposase InsAB	0.25	**4.04**
3361366	periplasmic chaperone protein	*yhcA*	>	*yhcD*	>	*ychE_1*	predicted protein, N-ter fragmer	0.25	**4.02**
272070	ed by insertion of IS30 element	*insO-1*	>		>	*ykfC*	CP4-6 prophage; conserved pr	0.25	**4.02**
383568	predicted glucosyltransferase	*yaiP*	<	*yaiS*	>	*tauA*	TauA	0.25	**4.01**
276360	MmuP APC transporter	*mmuP*	>	*mmuM*	<	*afuC*	CP4-6 prophage; predicted ferr	0.25	**4.01**
1201756	14 prophage; predicted protein	*ymfJ*	<	*ymfK*	>	*ymfT*	e14 prophage; predicted DNA-	0.25	**4.00**
2754968		*ssrA*	>	*intA*	<	*yfjH*	CP4-57 prophage; predicted pr	0.25	**4.00**
804848	YbhI DASS Transporter	*ybhI*	>	*ybhJ*	<	*ybhC*	outer membrane lipoprotein	0.25	**4.00**
1071042	dicted aminoacrylate hydrolase	*rutD*	<	*rutC*	<	*rutB*	peroxyureidoacrylate / ureidoac	0.25	**3.99**
1454070	ylacetyl-CoA epoxidase subunit	*paaC*	>	*paaD*	>	*paaE*	predicted ring 1,2-phenylacetyl-	0.25	**3.99**
285854	ophage; predicted dehydratase	*yagF*	>	*yagG*	>	*yagH*	putative β-xylosidase	0.25	**3.98**
1294134	dine kinase/deoxyuridine kinase	*tdk*	>	*ychG_2*	<	*ychG_1*	predicted protein, N-terminal fra	0.25	**3.98**
2075060	-44 prophage; predicted protein	*yeeT*	>		>	*yeeU*	CP4-44 prophage; antitoxin of t	0.25	**3.98**
586868	EnvY transcriptional activator	*envY*	<	*ybcH*	<	*nfrA*	bacteriophage N4 receptor, out	0.25	**3.96**

Chip-chip assay was performed for *E. coli* K-12 wild-type and its *rpoZ*-defective mutant strains. After tilling array analysis, the level of RNAP binding was compared between two strains. The ratio of binding levels between RpoZ-defective RNAP and wild-type RNAP was calculated for the entire probe along the genome, and is aligned in the decreasing order in the *rpoZ* column (shown in bold). Top 30 sites of incresed distributionl of the RpoZ-defective RNAP are described in Table 1.
