# Supplementary material for: Altered Distribution of RNA Polymerase Lacking the Omega Subunit within the Prophages along the Escherichia coli K-12 Genome
Source: mSystems. 2018 Feb 13;3(1):e00172-17. doi: 10.1128/mSystems.00172-17 (PMC5811629; doi:10.1128/mSystems.00172-17)
Supplement: TABLE S3 [file sys001182181st3.pdf]

Supplemental Table S3
Decreased level of distribution of the *rpoZ* mutant RNAP

Position	Function	Left	D	RNAP site	D	Right	Function	WT	***rpoZ***
293446	prophage; DNA-binding protein	*yagL*	<	*yagM*	<	*yagN*	CP4-6 prophage; predicted prot	37.2	**0.03**
2558866	5 prophage; predicted integrase	*intZ*	>	*yffL*	>	*yffM*	CPZ-55 prophage; predicted pr	32.5	**0.03**
293168	prophage; DNA-binding protein	*yagL*	<		<	*yagM*	CP4-6 prophage; predicted prot	21.5	**0.05**
2103636	predicted acyl transferase	*wbbJ*	<	*wbbI*	<	*rfc*	O-antigen polymerase	20.7	**0.05**
279962	-6 prophage; conserved protein	*yagB*	<		<	*yagA*	CP4-6 prophage; predicted DN/	19.7	**0.05**
1687138	fumarase A monomer	*fumA*	<	*manA*	>	*ydgA*	conserved protein	18.6	**0.05**
296336	age; predicted phage integrase	*intF*	<		<	*yagP*	predicted transcriptional regulat	17.0	**0.06**
284632	ophage; predicted dehydratase	*yagF*	>		>	*yagG*	YagG GPH Transporter	16.6	**0.06**
278132	component of ABC superfamily	*afuC*	<	*afuB*	<	*insAB-2*	InsAB' transposase	15.9	**0.06**
2298346	ferredoxin-type protein	*napG*	<	*napA*	<	*napD*	signal peptide-binding chaperon	15.3	**0.07**
2106448	UDP-galactopyranose mutase	*glf*	<	*rfbX*	<	*rfbC*	dTDP-4-dehydrorhamnose 3,5-	15.2	**0.07**
2188970	outer membrane usher protein	*yehB*	<		<	*yehC*	putative fimbrial chaperone	14.7	**0.07**
3334932	binding transcriptional regulator	*yrbA*	<		<	*mlaB*	MlaB	14.0	**0.07**
1201330	e14 prophage; predicted protein	*ymfJ*	<		<	*ymfK*	e14 prophage; predicted repres	13.6	**0.07**
3192540	putative membrane protein	*yqiK*	>		<	*sibD*	SibD small RNA	12.2	**0.08**
4620444	DeoD	*deoD*	>	*yjjJ*	<	*lplA*	lipoyl-protein ligase A	12.1	**0.08**
1429566	redicted protein, C-ter fragment	*lomR_2*	>	*stfR*	>	*tfaR*	Rac prophage; predicted tail fib	12.1	**0.08**
4200248	zinc homeostasis protein	*zraP*	<	*zraS*	>	*zraR*	ZraR transcriptional activator	12.1	**0.08**
2784038		*Phantom Ger*	<		>	*ygaQ_1*	predicted protein	11.8	**0.08**
279646	predicted IS protein	*ykgN*	<	*yagB*	<	*yagA*	CP4-6 prophage; predicted DN/	11.8	**0.08**
279738	predicted IS protein	*ykgN*	<	*yagB*	<	*yagA*	CP4-6 prophage; predicted DN/	11.5	**0.09**
1544666	predicted lipoprotein	*yddL*	<	*yddG*	>	*fdnG*	formate dehydrogenase N, &alp	11.4	**0.09**
2558272	5 prophage; predicted integrase	*intZ*	>		>	*yffL*	CPZ-55 prophage; predicted pr	11.2	**0.09**
2106534	UDP-galactopyranose mutase	*glf*	<	*rfbX*	<	*rfbC*	dTDP-4-dehydrorhamnose 3,5-	11.2	**0.09**
1428072	redicted protein, C-ter fragment	*lomR_2*	>	*stfR*	>	*tfaR*	Rac prophage; predicted tail fib	10.8	**0.09**
3678430	2-keto-3-deoxygluconokinase	*kdgK*	>		<	*yhjJ*	predicted zinc-dependent pepti	10.7	**0.09**
4473768	toxin-antitoxin biofilm protein	*tabA*	>	*yjgL*	<	*argI*	ArgI	10.7	**0.09**
3803272	haride core biosynthesis protein	*rfaS*	<	*waaP*	<	*waaG*	lipopolysaccharide glucosyltran	10.5	**0.09**
294058	4-6 prophage; predicted protein	*yagM*	<		<	*yagN*	CP4-6 prophage; predicted prot	10.4	**0.10**
2765968	-57 prophage; predicted protein	*yfjO*	>	*yfjP*	>	*yfjQ*	CP4-57 prophage; predicted pr	10.3	**0.10**
3467430	rioferritin-associated ferredoxin	*bfd*	<	*chiA*	<	*tufA*	elongation factor Tu	10.2	**0.10**
1311170	edicted inner membrane protein	*yciB*	<	*yciC*	>	*ompW*	OmpW, outer membrane protei	10.2	**0.10**
1736466	small membrane protein	*ynhF*	<	*purR*	<	*ydhB*	protein with similarity to transcri	10.1	**0.10**
293538	prophage; DNA-binding protein	*yagL*	<	*yagM*	<	*yagN*	CP4-6 prophage; predicted prot	9.9	**0.10**
3802572	ide-1,6-D-galactosyltransferase	*waaB*	<	*rfaS*	<	*waaP*	lipopolysaccharide core heptos	9.8	**0.10**
288440	binding transcriptional regulator	*yagI*	<		<	*argF*	ArgF	9.8	**0.10**
293372	prophage; DNA-binding protein	*yagL*	<	*yagM*	<	*yagN*	CP4-6 prophage; predicted prot	9.6	**0.10**
277762	omocysteine methyltransferase	*mmuM*	>	*afuC*	<	*afuB*	AfuB	9.5	**0.11**
294942	4-6 prophage; predicted protein	*yagN*	<		<	*intF*	CP4-6 prophage; predicted pha	9.2	**0.11**
2104144	1,6-galactofuranosyltransferase	*wbbI*	<	*rfc*	<	*glf*	UDP-galactopyranose mutase	9.2	**0.11**
284570	ophage; predicted dehydratase	*yagF*	>		>	*yagG*	YagG GPH Transporter	8.6	**0.12**
2989166	predicted protein	*ygeF*	>		>	*ygeG*	predicted chaperone	8.6	**0.12**
2106658	UDP-galactopyranose mutase	*glf*	<	*rfbX*	<	*rfbC*	dTDP-4-dehydrorhamnose 3,5-	8.6	**0.12**
293230	prophage; DNA-binding protein	*yagL*	<	*yagM*	<	*yagN*	CP4-6 prophage; predicted prot	8.5	**0.12**
277970	omocysteine methyltransferase	*mmuM*	>	*afuC*	<	*afuB*	AfuB	8.5	**0.12**
3798032	V; probably hexose transferase	*waaK*	<	*rfaZ*	<	*waaY*	lipopolysaccharide core heptos	8.4	**0.12**
2993554	predicted protein	*ygeL*	<	*ygeM*	<	*ygeN*	predicted protein	8.3	**0.12**
296170	4-6 prophage; predicted protein	*yagN*	<	*intF*	<	*yagP*	predicted transcriptional regulat	8.1	**0.12**
676832	predicted protein	*ybeR*	>	*djlB*	<	*ybeT*	conserved outer membrane pro	7.9	**0.13**
4429942	alanine/glycine APC transporter	*cycA*	>	*ytfE*	<	*ytfF*	inner membrane protein	7.9	**0.13**
546150	ureidoglycolate dehydrogenase	*allD*	<	*fdrA*	>	*ylbE_1*	predicted protein, N-ter fragmer	7.9	**0.13**
280064	-6 prophage; conserved protein	*yagB*	<		<	*yagA*	CP4-6 prophage; predicted DN/	7.9	**0.13**
294834	4-6 prophage; predicted protein	*yagN*	<		<	*intF*	CP4-6 prophage; predicted pha	7.8	**0.13**
3800232	α-1,2-glucosyltransferase	*waaJ*	<	*waaI*	<	*waaB*	UDP-D-galactose:(glucosyl)lipo	7.7	**0.13**
293040	-6 prophage; conserved protein	*yagK*	<	*yagL*	<	*yagM*	CP4-6 prophage; predicted prot	7.7	**0.13**
2562360	-55 prophage; predicted protein	*yffQ*	>	*yffR*	>	*yffS*	CPZ-55 prophage; predicted pr	7.7	**0.13**
1199836	phage exclusion; e14 prophage	*lit*	>	*intE*	<	*xisE*	e14 prophage; predicted excisio	7.6	**0.13**
3796640	O-antigen ligase	*rfaL*	>	*waaK*	<	*rfaZ*	protein involved in KdoIII attach	7.5	**0.13**
2101934	ynthesis protein, N-ter fragment	*wbbL_1*	<	*wbbK*	<	*wbbJ*	predicted acyl transferase	7.4	**0.13**

294432	4-6 prophage; predicted protein	*yagM*	<	*yagN*	<	*intF*	CP4-6 prophage; predicted pha	7.4	**0.14**
291460	4-6 prophage; predicted protein	*yagJ*	>		<	*yagK*	CP4-6 prophage; conserved pr	7.4	**0.14**
2104264	1,6-galactofuranosyltransferase	*wbbI*	<	*rfc*	<	*glf*	UDP-galactopyranose mutase	7.3	**0.14**
1596458	predicted lipoprotein	*ydeK*	<		<	*lsrK*	autoinducer-2 kinase	7.3	**0.14**
294332	4-6 prophage; predicted protein	*yagM*	<		<	*yagN*	CP4-6 prophage; predicted prot	7.2	**0.14**
576232	n protein; locus of qsr prophage	*nmpC*	<		>	*essD*	DLP12 prophage; predicted pha	7.1	**0.14**
3802172	ide-1,6-D-galactosyltransferase	*waaB*	<		<	*rfaS*	lipopolysaccharide core biosynt	7.1	**0.14**
1639072	phage; predicted S lysis protein	*essQ*	<		<	*cspB*	Qin prophage; cold shock prote	7.0	**0.14**
2767872	-57 prophage; predicted protein	*yfjQ*	>	*yfjR*	>	*ypjK*	CP4-57 prophage; predicted inn	7.0	**0.14**
2467506	actoprenyl glucosyl transferase	*yfdH*	>	*yfdI*	>	*tfaS*	CPS-53 (KpLE1) prophage; tail	7.0	**0.14**
4444030	outer membrane protein	*ytfM*	>	*ytfN*	>	*ytfP*	conserved protein	7.0	**0.14**
4091072	FrvA	*frvA*	<		<	*rhaM*	L-rhamnose mutarotase	7.0	**0.14**
4620636	DeoD	*deoD*	>	*yjjJ*	<	*lplA*	lipoyl-protein ligase A	6.9	**0.14**
292530	-6 prophage; conserved protein	*yagK*	<	*yagL*	<	*yagM*	CP4-6 prophage; predicted prot	6.9	**0.15**
287836	putative β-xylosidase	*yagH*	>	*yagI*	<	*argF*	ArgF	6.9	**0.15**
2382656	deubiquitinase	*elaD*	>	*yfbK*	>	*yfbL*	predicted peptidase	6.9	**0.15**
1637130	in prophage; cold shock protein	*cspI*	<	*ydfP*	<	*ydfQ*	Qin prophage; predicted lysozy	6.8	**0.15**
284342	hage; predicted lyase/synthase	*yagE*	>	*yagF*	>	*yagG*	YagG GPH Transporter	6.8	**0.15**
4093968	1-phosphate aldolase monomer	*rhaD*	<	*rhaA*	<	*rhaB*	L-rhamnulose kinase	6.8	**0.15**
2556732	mmonia-lyase, α subunit	*eutB*	<		>	*intZ*	CPZ-55 prophage; predicted int	6.8	**0.15**
3800158	α-1,2-glucosyltransferase	*waaJ*	<	*waaI*	<	*waaB*	UDP-D-galactose:(glucosyl)lipo	6.7	**0.15**
4568572	predicted transposase	*yjiQ*	>	*yjiR*	>	*yjiS*	conserved protein	6.7	**0.15**
2993746	predicted protein	*ygeM*	<		<	*ygeN*	predicted protein	6.7	**0.15**
2763154	-57 prophage; predicted protein	*yfjL*	<		<	*yfjM*	CP4-57 prophage; predicted pr	6.7	**0.15**
2185930	predicted protein	*yohN*	>	*yehA*	<	*yehB*	putative outer membrane usher	6.6	**0.15**
284448	ophage; predicted dehydratase	*yagF*	>		>	*yagG*	YagG GPH Transporter	6.6	**0.15**
2472336	E1) prophage; predicted protein	*yfdP*	>	*yfdQ*	>	*yfdR*	CPS-53 (KpLE1) prophage; dec	6.6	**0.15**
1644936	Qin prophage; predicted protein	*flxA*	>		<	*ydfW*	Qin prophage; predicted protein	6.5	**0.15**
2190388	ted fimbrial-like adhesin protein	*yehD*	<		<	*yehE*	predicted protein	6.5	**0.15**
3095868		*rdgB*	>	*yggW*	<	*yggM*	conserved protein	6.4	**0.16**
2464466	tRNA^{argW}	*argW*	>		>	*intS*	CPS-53 (KpLE1) prophage; pro	6.4	**0.16**
2558668	5 prophage; predicted integrase	*intZ*	>	*yffL*	>	*yffM*	CPZ-55 prophage; predicted pr	6.4	**0.16**
1647062	Qin prophage; predicted protein	*rzpQ*	>		>	*dicF*	Qin prophage; DicF antisense F	6.3	**0.16**
575258		*insH-2*	<	*nmpC*	>	*essD*	DLP12 prophage; predicted pha	6.3	**0.16**
2559738	-55 prophage; predicted protein	*yffM*	>	*yffN*	>	*yffO*	CPZ-55 prophage; predicted pr	6.3	**0.16**
4570170	conserved protein	*yjiS*	>		>	*yjiT*	conserved protein	6.2	**0.16**
295350	4-6 prophage; predicted protein	*yagN*	<	*intF*	<	*yagP*	predicted transcriptional regulat	6.2	**0.16**
4572350	conserved protein	*yjiT*	>	*yjiV*	<	*mcrC*	MrcC subunit of 5-methylcytosin	6.2	**0.16**
3359930	tamate synthase, small subunit	*gltD*	>	*gltF*	>	*yhcA*	predicted periplasmic chaperon	6.1	**0.16**
127764	ydrolipoamide acetyltransferase	*aceF*	>		>	*lpd*	E3 monomer	6.1	**0.16**
2882562	Cascade subunit A	*casA*	<		<	*cas3*	predicted fused HD nuclease an	6.1	**0.17**
2779238	YpjF-YfjZ toxin-antitoxin system	*ypjF*	>	*ypjA*	<	*pinH*	predicted invertase fragment	6.0	**0.17**
4089562	frv operon protein	*frvX*	<	*frvB*	<	*frvA*	FrvA	6.0	**0.17**
2601070	hydrogenase 4, component A	*hyfA*	>	*hyfB*	>	*hyfC*	hydrogenase 4, component C	6.0	**0.17**
1463152	eptide repeat acetyltransferase	*paaY*	>		<	*ynbG*	small protein	5.9	**0.17**
3801168	α-1,3-glucosyltransferase	*waaI*	<	*waaB*	<	*rfaS*	lipopolysaccharide core biosynt	5.8	**0.17**
294172	4-6 prophage; predicted protein	*yagM*	<		<	*yagN*	CP4-6 prophage; predicted prot	5.7	**0.17**
4435856	'),5'-bisphosphate nucleotidase	*cysQ*	>	*ytfI*	<	*ytfJ*	conserved protein	5.7	**0.18**
4570068	conserved protein	*yjiS*	>		>	*yjiT*	conserved protein	5.7	**0.18**
2070240	IsrC small RNA	*isrC*	>	*flu*	>	*yeeR*	CP4-44 prophage; predicted me	5.6	**0.18**
293632	prophage; DNA-binding protein	*yagL*	<	*yagM*	<	*yagN*	CP4-6 prophage; predicted prot	5.6	**0.18**
4313832	-CoA N-acyltransferase domain	*phnO*	<	*phnN*	<	*phnM*	ATP-binding component of phos	5.6	**0.18**
2103554	predicted acyl transferase	*wbbJ*	<	*wbbI*	<	*rfc*	O-antigen polymerase	5.6	**0.18**
789330	galactokinase	*galK*	<	*galT*	<	*galE*	UDP-glucose 4-epimerase mon	5.5	**0.18**
2031954	of OmpA and OmpC expression	*rseX*	>		>	*yedS_1*	predicted protein, N-ter fragmen	5.5	**0.18**
2993634	predicted protein	*ygeL*	<	*ygeM*	<	*ygeN*	predicted protein	5.5	**0.18**
2879966	Cascade subunit D	*casD*	<	*casC*	<	*casB*	Cascade subunit B	5.5	**0.18**
2108132	polysaccharide PST transporter	*rfbX*	<	*rfbC*	<	*rfbA*	dTDP-glucose pyrophosphoryla	5.4	**0.18**
292040	4-6 prophage; predicted protein	*yagJ*	>	*yagK*	<	*yagL*	CP4-6 prophage; DNA-binding	5.4	**0.18**
2882356	Cascade subunit A	*casA*	<		<	*cas3*	predicted fused HD nuclease an	5.4	**0.19**
2185770	predicted protein	*yohN*	>	*yehA*	<	*yehB*	putative outer membrane usher	5.4	**0.19**
1588350	predicted protein	*ydeT*	<		<	*yneL*	predicted transcriptional regulat	5.4	**0.19**

3800334	α-1,2-glucosyltransferase	*waaJ*	<	*waaI*	<	*waaB*	UDP-D-galactose:(glucosyl)lipo	5.4	**0.19**
2386672	predicted protein	*yfbN*	<	*yfbO*	>	*yfbP*	predicted protein	5.4	**0.19**
2885168	Cascade subunit A	*casA*	<	*cas3*	>	*sokX*	SokX	5.3	**0.19**
3805730	accharide glucosyltransferase I	*waaG*	<	*waaQ*	>	*waaA*	KDO transferase	5.3	**0.19**
2234632	imidine dehydrogenase subunit	*yeiA*	>		<	*mglC*	MglC	5.2	**0.19**
2755760	CP4-57 prophage; integrase	*intA*	>	*yfjH*	>	*alpA*	AlpA transcriptional activator; C	5.2	**0.19**
1470046	transposase of IS30	*insI-2*	>	*ydbA_2*	>	*ydbC*	predicted oxidoreductase, NAD	5.2	**0.19**
2992670	peptidoglycan-binding enzyme	*pbl*	>	*ygeK*	<	*ygeL*	predicted protein	5.2	**0.19**
1468846	transposase of IS30	*insI-2*	>	*ydbA_2*	>	*ydbC*	predicted oxidoreductase, NAD	5.2	**0.19**
296234	4-6 prophage; predicted protein	*yagN*	<	*intF*	<	*yagP*	predicted transcriptional regulat	5.2	**0.19**
636048	conserved protein	*ybdN*	<	*ybdO*	<	*dsbG*	DsbG_{reduced}	5.2	**0.19**
2765266	CP4-57 prophage; RNase LS	*rnlA*	>	*yfjO*	>	*yfjP*	CP4-57 prophage; predicted G	5.2	**0.19**
3416664	ted outer membrane lipoprotein	*yhdV*	>		>	*yhdW*	YhdW	5.2	**0.19**
1095368	predicted protein	*ymdE*	>	*ycdU*	<	*serX*	tRNA^{serX}	5.2	**0.19**
1643734	ce the repressor activity of RelB	*relE*	<	*relB*	>	*ydfV*	Qin prophage; predicted protein	5.1	**0.19**
1635936	Qin prophage; predicted protein	*gnsB*	<		<	*ynfN*	Qin prophage; predicted protein	5.1	**0.20**
3796746	O-antigen ligase	*rfaL*	>	*waaK*	<	*rfaZ*	protein involved in KdoIII attach	5.1	**0.20**
2992436	peptidoglycan-binding enzyme	*pbl*	>		<	*ygeK*	predicted DNA-binding transcrip	5.1	**0.20**
2765354	-57 prophage; predicted protein	*yfjO*	>		>	*yfjP*	CP4-57 prophage; predicted G	5.1	**0.20**
238730	C0067 small RNA	*C0067*	>		<	*yafU*	predicted inner membrane prote	5.0	**0.20**
2387730	predicted protein	*yfbO*	>	*yfbP*	<	*nuoN*	NADH:ubiquinone oxidoreducta	5.0	**0.20**
2988170	small protein	*yqeL*	<	*yqeK*	>	*ygeF*	predicted protein	5.0	**0.20**
927556	alanyl-tRNA-protein transferase	*aat*	<	*cydC*	<	*cydD*	CydD	5.0	**0.20**
2102040	ynthesis protein, N-ter fragment	*wbbL_1*	<	*wbbK*	<	*wbbJ*	predicted acyl transferase	5.0	**0.20**
3806064	accharide glucosyltransferase I	*waaG*	<	*waaQ*	>	*waaA*	KDO transferase	4.9	**0.20**
2559956	-55 prophage; predicted protein	*yffM*	>	*yffN*	>	*yffO*	CPZ-55 prophage; predicted pr	4.9	**0.20**
3806372	de core heptosyl transferase III	*waaQ*	<		>	*waaA*	KDO transferase	4.9	**0.20**
2562046	-55 prophage; predicted protein	*yffQ*	>	*yffR*	>	*yffS*	CPZ-55 prophage; predicted pr	4.9	**0.20**
2189932	putative fimbrial chaperone	*yehC*	<	*yehD*	<	*yehE*	predicted protein	4.9	**0.20**
2105234	O-antigen polymerase	*rfc*	<		<	*glf*	UDP-galactopyranose mutase	4.9	**0.20**
2988770	predicted protein	*yqeK*	<	*ygeF*	>	*ygeG*	predicted chaperone	4.9	**0.20**
3036248	RecJ	*recJ*	<	*dsbC*	<	*xerD*	site-specific recombinase	4.9	**0.20**
1471130	transposase of IS30	*insI-2*	>	*ydbA_2*	>	*ydbC*	predicted oxidoreductase, NAD	4.9	**0.20**
3700368	YhjV STP transporter	*yhjV*	>	*dppF*	<	*dppD*	DppD	4.9	**0.21**
2559840	-55 prophage; predicted protein	*yffM*	>	*yffN*	>	*yffO*	CPZ-55 prophage; predicted pr	4.9	**0.21**
2106830	UDP-galactopyranose mutase	*glf*	<	*rfbX*	<	*rfbC*	dTDP-4-dehydrorhamnose 3,5-	4.8	**0.21**
582934	P12 prophage; predicted protein	*tfaX*	>	*appY*	<	*ompT*	outer membrane protease VII (	4.8	**0.21**
1991670	tidylglycerophosphate synthase	*pgsA*	<	*uvrC*	<	*uvrY*	UvrY transcriptional regulator	4.8	**0.21**
3360048	periplasmic protein	*gltF*	>		>	*yhcA*	predicted periplasmic chaperon	4.8	**0.21**
238854	C0067 small RNA	*C0067*	>	*yafU*	>	*yafF*	conserved protein	4.8	**0.21**
2558970	-55 prophage; predicted protein	*yffL*	>		>	*yffM*	CPZ-55 prophage; predicted pr	4.8	**0.21**
2838064	AscG transcriptional repressor	*ascG*	<	*ascF*	>	*ascB*	6-phospho-β-glucosidase;	4.8	**0.21**
2878270	predicted integrase	*cas1*	<	*casE*	<	*casD*	Cascade subunit D	4.7	**0.21**
292656	-6 prophage; conserved protein	*yagK*	<	*yagL*	<	*yagM*	CP4-6 prophage; predicted prot	4.7	**0.21**
4057356	GlnA	*glnA*	<	*typA*	>	*yihL*	YihL putative transcriptional reg	4.7	**0.21**
2878936	crRNA endonuclease	*casE*	<	*casD*	<	*casC*	Cascade subunit C	4.7	**0.21**
2988240	small protein	*yqeL*	<	*yqeK*	>	*ygeF*	predicted protein	4.6	**0.22**
291064		*insA-3*	<	*yagJ*	<	*yagK*	CP4-6 prophage; conserved pro	4.6	**0.22**
2406734	hA transcriptional dual regulator	*lrhA*	<	*yfbQ*	>	*yfbR*	dCMP phosphohydrolase	4.6	**0.22**
2467692	actoprenyl glucosyl transferase	*yfdH*	>	*yfdI*	>	*tfaS*	CPS-53 (KpLE1) prophage; tail	4.6	**0.22**
1633262	licted tail fibre assembly protein	*tfaQ*	<	*ydfN*	<	*nohA*	Qin prophage; predicted packag	4.6	**0.22**
2101034		*insH-7*	<	*wbbL_1*	<	*wbbK*	predicted lipopolysaccharide bio	4.6	**0.22**
673772	rare lipoprotein LptE	*lptE*	<	*leuS*	>	*ybeL*	conserved protein	4.6	**0.22**
2763338	-57 prophage; predicted protein	*yfjL*	<		<	*yfjM*	CP4-57 prophage; predicted pro	4.6	**0.22**
2558062	5 prophage; predicted integrase	*intZ*	>		>	*yffL*	CPZ-55 prophage; predicted pr	4.6	**0.22**
4436036	'),5'-bisphosphate nucleotidase	*cysQ*	>	*ytfI*	<	*ytfJ*	conserved protein	4.5	**0.22**
2103932	predicted acyl transferase	*wbbJ*	<	*wbbI*	<	*rfc*	O-antigen polymerase	4.5	**0.22**
2101836	ynthesis protein, N-ter fragment	*wbbL_1*	<	*wbbK*	<	*wbbJ*	predicted acyl transferase	4.5	**0.22**
292260	-6 prophage; conserved protein	*yagK*	<		<	*yagL*	CP4-6 prophage; DNA-binding	4.5	**0.22**
2988054	small protein	*yqeL*	<	*yqeK*	>	*ygeF*	predicted protein	4.5	**0.22**
3801248	α-1,3-glucosyltransferase	*waaI*	<	*waaB*	<	*rfaS*	lipopolysaccharide core biosynt	4.5	**0.22**
3798254	olysaccharide core biosynthesis	*rfaZ*	<		<	*waaY*	lipopolysaccharide core heptos	4.5	**0.22**

2252332	rimidine nucleoside transporter	*nupX*	<	*rihB*	>	*yeiL*	YeiL transcriptional activator	4.5	**0.22**
2140654	Dcd	*dcd*	<	*udk*	>	*yegE*	predicted diguanylate cyclase	4.5	**0.22**
2385530	predicted protein	*yfbM*	>		<	*yfbN*	predicted protein	4.4	**0.22**
265132	4-6 prophage; predicted protein	*ykfF*	<	*ykfB*	<	*yafY*	CP4-6 prophage; inner membra	4.4	**0.23**
3805672	accharide glucosyltransferase I	*waaG*	<	*waaQ*	>	*waaA*	KDO transferase	4.4	**0.23**
2987942	small protein	*yqeL*	<		<	*yqeK*	predicted protein	4.4	**0.23**
798130	se/fructose 1,6-bisphosphatase	*ybhA*	<	*pgl*	<	*ybhD*	predicted DNA-binding transcri	4.4	**0.23**
280360	-6 prophage; conserved protein	*yagB*	<	*yagA*	>	*yagE*	CP4-6 prophage; predicted lyas	4.4	**0.23**
2069334	; predicted GTP-binding protein	*yeeP*	>		>	*isrC*	IsrC small RNA	4.4	**0.23**
1643558	ophage; small toxic polypeptide	*hokD*	<	*relE*	<	*relB*	RelB Qin prophage; antitoxin of	4.4	**0.23**
2878344	predicted integrase	*cas1*	<	*casE*	<	*casD*	Cascade subunit D	4.4	**0.23**
3800548	α-1,2-glucosyltransferase	*waaJ*	<	*waaI*	<	*waaB*	UDP-D-galactose:(glucosyl)lipo	4.3	**0.23**
2987772	edicted transcriptional regulator	*yqeI*	>	*yqeJ*	<	*yqeL*	small protein	4.3	**0.23**
3803540	haride core biosynthesis protein	*rfaS*	<	*waaP*	<	*waaG*	lipopolysaccharide glucosyltran	4.3	**0.23**
2107634	polysaccharide PST transporter	*rfbX*	<		<	*rfbC*	dTDP-4-dehydrorhamnose 3,5-	4.3	**0.23**
2385772	predicted protein	*yfbM*	>	*yfbN*	>	*yfbO*	predicted protein	4.3	**0.23**
2763272	-57 prophage; predicted protein	*yfjL*	<		<	*yfjM*	CP4-57 prophage; predicted pr	4.3	**0.23**
2755870	CP4-57 prophage; integrase	*intA*	>	*yfjH*	>	*alpA*	AlpA transcriptional activator; C	4.3	**0.24**
586152	EnvY transcriptional activator	*envY*	<		<	*ybcH*	predicted protein	4.3	**0.24**
2107730	polysaccharide PST transporter	*rfbX*	<	*rfbC*	<	*rfbA*	dTDP-glucose pyrophosphoryla	4.2	**0.24**
3800958	α-1,2-glucosyltransferase	*waaJ*	<	*waaI*	<	*waaB*	UDP-D-galactose:(glucosyl)lipo	4.2	**0.24**
576360	n protein; locus of qsr prophage	*nmpC*	<		>	*essD*	DLP12 prophage; predicted pha	4.2	**0.24**
273772	-6 prophage; conserved protein	*ykfC*	>	*insH-1*	>	*mmuP*	MmuP APC transporter	4.2	**0.24**
1646036	anscriptional regulator for DicB	*dicC*	<	*dicA*	>	*ydfA*	Qin prophage; predicted protei	4.2	**0.24**
3803124	haride core biosynthesis protein	*rfaS*	<		<	*waaP*	lipopolysaccharide core heptos	4.2	**0.24**
1461572	phenylacetate-CoA ligase	*paaK*	>		>	*paaX*	PaaX	4.2	**0.24**
4395832	muramoyl-L-alanine amidase 2	*amiB*	>	*mutL*	>	*miaA*	tRNA(i⁶A37) synth	4.2	**0.24**
1599036	autoinducer-2 kinase	*lsrK*	<	*lsrR*	>	*lsrA*	LsrA	4.2	**0.24**
1644664	Qin prophage; predicted protein	*ydfV*	>	*flxA*	<	*ydfW*	Qin prophage; predicted protei	4.1	**0.24**
58658	chaperone with DnaK	*djlA*	>	*yabP*	>	*yabQ*	predicted protein	4.1	**0.24**
1645350	Qin prophage; predicted protein	*ydfW*	<		<	*ydfX*	Qin prophage; predicted protei	4.1	**0.24**
3796534	O-antigen ligase	*rfaL*	>	*waaK*	<	*rfaZ*	protein involved in KdoIII attach	4.1	**0.24**
3780254	predicted rRNA methylase	*yibK*	>	*cysE*	<	*gpsA*	GpsA	4.1	**0.24**
4570370	conserved protein	*yjiS*	>		>	*yjiT*	conserved protein	4.1	**0.24**
1633630	licted tail fibre assembly protein	*tfaQ*	<	*ydfN*	<	*nohA*	Qin prophage; predicted packa	4.1	**0.25**
690530	polipoprotein N-acyltransferase	*lnt*	<	*ybeX*	<	*ybeY*	conserved protein involved in tr	4.1	**0.25**
2092660		*hisB*	>	*hisH*	>	*hisA*	N-(5'-phospho-L-ribosyl-formim	4.0	**0.25**
4476964	bonuclease E inhibitor protein B	*rraB*	>		<	*yjgM*	predicted acetyltransferase	4.0	**0.25**
2988370	predicted protein	*yqeK*	<		>	*ygeF*	predicted protein	4.0	**0.25**
2845070	hydrogenase 3, large subunit	*hycE*	<	*hycD*	<	*hycC*	hydrogenase 3, membrane sub	4.0	**0.25**
3799872	charide core heptose (II) kinase	*waaY*	<	*waaJ*	<	*waaI*	UDP-D-glucose:(glucosyl)LPS	4.0	**0.25**
2763970	-57 prophage; predicted protein	*yfjM*	<	*rnlA*	>	*yfjO*	CP4-57 prophage; predicted pr	4.0	**0.25**
2387644	predicted protein	*yfbO*	>	*yfbP*	<	*nuoN*	NADH:ubiquinone oxidoreducta	4.0	**0.25**

Chip-chip assay was performed for *E. coli* K-12 wild-type and its *rpoZ*-defective mutant strains. After tilling array analysis, the level of RNAP binding was compared between two strains. The ratio between the biding of RpoZ-defective RNAP and that of wild-type RNAP was calculated for the entire probe along the genome. This value is aligned in the increasing order in the *rpoZ* column (shown in bold). Top 31genes of low-level distribution of the RpoZ-defective RNAP are described in Table 2.
